# Optimized Artificial Colonic Mucus Enabling Physiologically Relevant Diffusion Studies of Drugs, Particles, and Delivery Systems

**DOI:** 10.1021/acs.molpharmaceut.5c00298

**Published:** 2025-06-10

**Authors:** Marco Tjakra, Nopdanai Chakrapeesirisuk, Magdalena Jacobson, Mikael E. Sellin, Jens Eriksson, Alexandra Teleki, Christel A. S. Bergström

**Affiliations:** † Department of Pharmacy, Uppsala Biomedical Center, 8097 Uppsala University, 751 23 Uppsala, Sweden; ‡ The Swedish Drug Delivery Center, Department of Pharmacy, 8097 Uppsala University, Box 580, SE-751 6 23 Uppsala, Sweden; § Department of Pharmacy, Science for Life Laboratory, 8097 Uppsala University, 751 23 Uppsala, Sweden; ∥ Department of Clinical Sciences, Faculty of Veterinary Medicine and Animal Science, 8095 Swedish University of Agricultural Sciences, Box 7054, SE-750 07 Uppsala, Sweden; ⊥ Department of Medical Biochemistry and Microbiology, Uppsala University, 751 23 Uppsala, Sweden; # Science for Life Laboratory, 751 23 Uppsala, Sweden

**Keywords:** mucus, hydrogel, drug, diffusion, rheology, binding, structure, drug delivery, colon

## Abstract

Development of oral drug delivery systems that penetrate the colonic mucus remains challenging. Artificial models of porcine colonic mucus have been developed that mimic the rheology and viscosity of the native mucus and its contents of mucins, protein, and lipids. However, they are less representative with regard to the zeta potential, a factor of importance for charged molecules and particles. This study therefore aimed to improve the existing porcine artificial colonic mucus model by exchanging the polymer backbone (used for viscosity) to more closely mimic the charge of porcine native colonic mucus. Polymers studied were poly­(acrylic acid), hydroxyethylcellulose, sodium hyaluronate, sodium alginate, and pectin. The resulting porcine artificial colonic mucus was assayed for apparent viscosity, storage modulus, pH, water content, zeta potential, and pore size. The two best-performing polymers (poly­(acrylic acid) and hydroxyethylcellulose) were then assayed with diffusion of FITC-dextran, particle tracking of nanoparticles, and binding of FITC-dextran and contrasted to data generated in porcine native colonic mucus (PNCM). Of the two polymers, PACM based on HEC generated zeta potential and binding kinetics similar to those of PNCM. We conclude that the choice of polymer in PACMs is critical for improving their use in drug development. The extensive characterization of the PACMs further points toward the importance of complementary techniques to determine rheological characteristics, mesh, and pore size.

## Graphical abstract

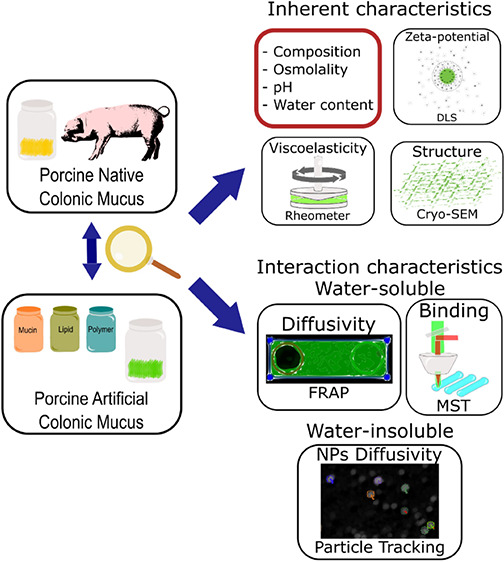

## Introduction

Oral drug delivery remains the most preferred route of administration for the advantages it confers on patient compliance. It typically targets absorption to the systemic circulation because of the large absorptive surface area of the small intestine.^1^ However, local release in and absorption from the colon has gained interest. The colonic region has a different physiology than the small intestine, having a thicker and more viscous mucus layer that hinders the diffusion and permeability of drugs to the epithelium.^2^ To improve the development process for drugs intended for absorption from the colon, a systematic and standardized experimental platform capable of capturing the physiology of the colon is critical to ensure reproducible and physiologically relevant measurements. Such a platform would need to include the mucus barrier; however, unfortunately, there is no consensus on which colonic mucus model to use for in vitro screening of drug diffusion and binding.

There are several approaches to investigate drug diffusion^3^ and binding in the mucus.^4^ Depending on the method, resourcing the mucus sample is the main challenge. Preclinical animal models serve as a mucus source but are typically limited by volume, ethical issues, and interindividual variability.^5^ Current attempts to modify cell culture for mucus secretion are limited by production cost, low mucus yield, and lower mucus quality with respect to viscosity.^6^ In response to these challenges, biosimilar artificial mucus models have been developed.^7^ One emerging preclinical species for drug delivery studies is pig. The pig colonic environment shares similarities to humans in terms of pH and bacterial flora,^8^ and in this study, we therefore evaluated porcine artificial colonic mucus as an in vitro model for drug studies of colonic drug delivery.

The FDA Modernization Act 2.0 highlights the potential of nonanimal testing and the introduction of biosimilar concept for drug testing.^9^ Artificial mucus is one of the new animal-free models that can be used, among others, to study drug diffusion. Artificial mucus is easy to produce, mimics the native porcine colonic mucus well, provides reproducibility, and is a low-cost alternative to more complex systems. However, there are challenges to producing an artificial mucus that resembles all of the characteristics of the native mucus and its role as a physical barrier. Commercially available purified mucins have lost their capacity to form a strong gel structure due to the loss of glycosylation.^10^ To compensate for this, studies have proposed the use of polymers to produce the highly viscous structure and functionality as a physical barrier.^11–13^ Hence, the combination of purified mucin and polymers would provide the binding interactions similar to those occurring in vivo and the physical structure. Drug diffusion through the hydrogel is affected by various factors, such as viscosity, pore size, zeta potential, and water content. Even though the inherent physicochemical properties of porcine artificial colonic mucus (PACM) are similar to those of the porcine native colonic mucus (PNCM), it is important to evaluate the physiological relevance of the PACM for drug delivery and absorption studies.

In this study, we therefore explored the effects of different polymers on viscosity, rheology, and zeta potential. The optimized PACM was thereafter used to study diffusion and binding of model compounds and particles for comparison with the PNCM, with the objective to investigate the physiological relevance for drug delivery and absorption studies.

## Materials and Methods

### Ethics Statement for Pig Colonic Mucus and Tissue Samples

Porcine native colonic mucus from pig colonic tissue was obtained from the local abattoir in Lövsta, Uppsala. Following the Uppsala University framework for animal study, no ethical permission was required for this study involving animal byproducts produced for commercial consumption. Personnel involved in the sampling activities have completed the laboratory animal science–pig course from Uppsala University, and all sampling activities were done under the supervision of a veterinarian. The sampling procedure followed a previous study.^14^

### Materials

Polyacrylic acid (PAA; Carbopol 974P NF) was purchased from Lubrizol (Brussels, Belgium). Sodium hyaluronate (SH) was purchased from ACROS Organics (Geel, Belgium). Hydroxyethylcellulose (HEC; Natrosol 250 HHX PHARM) was a gift from Ashland (Columbus, OH, USA). Sodium alginate (SA; from brown algae, medium viscosity), pectin (P; from apple), porcine mucin type II (from porcine stomach), bovine serum albumin (BSA), cholesterol, Tween 80, 2-(bis­(2-hydroxyethyl)­amino)­ethanesulfonic acid (BES), 2-(N-morpholino)­ethanesulfonic acid (MES), MgSO_4_·7H_2_O, and CaCl_2_ were purchased from Sigma-Aldrich (St. Louis, MO, USA). Phosphatidyl choline was procured as a gift from Lipoid GmbH (Ludwigshafen, Germany). Fluorescein isothiocyanate (FITC)–diethylaminoethyl (DEAE)-dextran with molecular weights 4000 (FD4K+) was obtained from Sigma-Aldrich. For negatively charged particles, latex beads (carboxylate-modified polystyrene) with sizes 0.1 (F8803), 0.2 (F8811), and 1 (F8823) μm were bought from Thermo Fisher Scientific and 0.5 μm (L3280) from Sigma-Aldrich. For the positively charged particle model, latex beads (amine-modified polystyrene) with 0.1 μm mean particle size were procured from Sigma-Aldrich (L9904), and for 0.2 and 1 μm sizes, they were procured from Thermo Fisher Scientific (F8764, F8765). 5 M sodium hydroxide and 5 M hydrochloric acid were used for pH adjustments.

### Polymer Selection

Formulations of the PACMs (Table S1) were adapted from previous work,^14^ with modifications of polymer type and concentration. PAA, the polymer used in the previously established PACM, was used as a comparison.

Polymer candidates were selected through literature review by considering their previous use in artificial mucus models and their likelihood of providing a (i) gel-like structure, (ii) net negative charge at colonic pH (pH 7.3–7.5 ^14^), and (iii) mesh microstructure. Polymers selected in this study were chosen to allow production of the artificial mucus in the common laboratory, and hence, we focused on polymers commercially available. From these criteria, five polymers were selected (Table 1). PAA and HEC have been previously proposed as suitable polymers for artificial mucus to mimic the mucus barrier of the porcine intestine^12^ and horse respiratory tract,^15^ respectively. The SH and porcine stomach mucin type III mixture has also been proposed, where the high-molecular-weight SH modulates mucin nanostructure and mesh size.^16^ SA has also been used to hinder diffusion in small intestinal mucus by providing barrier properties to mimic the mucus.^17^ Pectin mixed with mucin has been shown to enhance the mucin network through electrostatic interactions, resulting in increased viscosity.^18^

**Table 1:** Summary of Characteristics for the Porcine Native Colonic Mucus (PNCM) and the Artificial Mucus Model (PACM) with Four Different Polymer Backbones as the Gelling Agent^a^

sample	optimal concentration of gelling agent (w/v)	pH (*x̅* ± SD)	osmolality (mOsm/kg) (*x̅* ± SD)	water content (*x̅* ± SD) (%)	zeta potential (mV)	storage modulus in LVR (MPa)	viscoelastic gel-like behavior
PNCM	2–5% mucin^14^	7.3–7.5 ^14^	ND	89.4 ± 1.0 ^14^	–15 to −22	4.5 × 10^–5^ to 2.6 × 10^–2^	yes
PACMs in MES buffer	1.5% PAA	5.36 ± 0.04^b^	132 ± 1	90.1 ± 0.6	–45 to −55	2.8 × 10^–4^ to 4.1 × 10^–4^	yes
		7.14 ± 0.28^c^					
	5.0% HEC	6.30 ± 0.07	106 ± 2	88.9 ± 0.7	–17 to −20	5.5 × 10^–4^ to 2.1 × 10^–3^	yes
	3.0% SH	6.31 ± 0.03	139 ± 2	90.3 ± 1.4	–32 to −36	1.4 × 10^–4^ to 6.9 × 10^–4^	yes
	5.0% SA	6.35 ± 0.13	205 ± 1	85.5 ± 1.2	–49 to −51	5.0 × 10^–5^ to 8.9 × 10^–4^	no
PACM in BES buffer	3% HEC^d^	7.30 ± 0.07	134 ± 2	88.9 ± 0.7	–19 to −23	7.5 × 10^–5^ to 4.8 × 10^–4^	yes

a Abbreviations: PNCM—Porcine native colonic mucus; PAA—polyacrylic acid; HEC—hydroxyethyl cellulose; SH—sodium hyaluronate; SA—sodium alginate; Osmolality values were determined to avoid hyperosmolality (>372 mOsm/kg), which is cytotoxic to Caco-2 cells.^33^ This compatibility of the PACM with Caco-2 cells is important since absorption models simultaneously addressing the cell barrier and the mucus barrier are warranted.

b Before pH adjustment.

c After pH adjustment.

d Control of 1.5% PAA dissolved in BES resulted in a mean zeta potential of −40 to −47; ND: not determined.

### PACM Formulation

A first screening of the amount of polymer that was needed was determined through visual inspection by serial addition of the polymer (1–5% w/v) until a gel-like structure was obtained similar to that of the PACM PAA model. The suitable addition was confirmed by viscosity sweep and storage modulus measurement with targets to mimic PNCM values (Table 1).

In a second step, the polymer (HEC) that produced the zeta potential (for measurement settings, see below) similar to PNCM was selected and further optimized. In this stage, the buffer was changed to allow a pH of the PACM similar to that of the PNCM. MES was exchanged to N,N-bis­(2-hydroxyethyl)-2-aminoethanesulfonic acid (BES), and the HEC zeta potential was contrasted to the control (PACM PAA in BES).

In a third step, the concentration of HEC was optimized to produce interactions between the drug molecules and the mucus mesh similar to that observed in PNCM.

### Fresh Porcine Native Colonic Mucus Collection

Fresh native mucus was collected according to the protocol developed by Barmpatsalou et al.^14^ Briefly, the gastrointestinal tract (GIT) of crossbreed Landrace pigs (20–22 weeks, 100–110 kg) was collected from a local abattoir (*n* = 3). The pigs were fasted ≥12 h, with access to water prior to slaughter. Dissection began within 1 h after slaughter. The proximal colon was excised and removed from the remaining GIT and then gently rinsed twice in ice-cold isotonic buffer (10 mM MES buffer containing 1.3 mM CaCl2, 1.0 mM MgSO_4_, and 137 mM NaCl, pH 6.5) to eliminate solid contents. The rinsed tissue was pinned to styrofoam with syringe needles, and the mucus was gently collected using a laboratory spatula. The collected mucus was kept in a glass vial and stored in an ice bath directly after sampling. The fresh mucus was either used on the collection day or stored at −80 °C.

### Osmolality Measurement

Osmolality of PACMs was measured using a Fiske 210 Micro-Osmometer (Advanced Instruments, Norwood, MA, USA). This single-sample, freezing-point depression micro-osmometer used a sample size of approximately 100 μg. Experiments were performed in triplicate.

### Bulk Rheology and Theoretical Mesh Size

PACM was prepared 1 day before the rheological measurements. An ARES-G2 strain-controlled rheometer (TA Instruments, Delaware, USA) with an Advanced Peltier System (APS) accessory was assembled. A 25 mm diameter stainless steel upper geometry (0.0174 rad cone angle and 0.021 truncation gap) and a 60 mm diameter lower geometry with a hardened chromium surface and quick-change system were used. Visual observation after each measurement confirmed that samples are properly loaded in between geometries. The cone and plate geometry was selected to provide more grip on the sample and enable the minimum volume needed. Mucus sample rheology was profiled at 37 °C in a solvent trap to prevent evaporation. The apparent viscosity (η) of mucus samples was continuously measured by ramping the shear rate from 0.1 to 500 s-^1^. Then, the linear viscoelastic region (LVR) was identified in an amplitude sweep from oscillation strain 0.01 to 100.0% at a 1 Hz frequency. Finally, the storage (*G*′) and loss modulus (*G*″) were measured in a frequency sweep from angular frequency 0.63 to 60 rad/s at 0.5% oscillation strain. The settings were within the LVR to prevent structural damage, and 0.5% was chosen to accommodate both native and artificial mucus without getting to the lower limit of detection. The rheological profiles of the artificial mucus samples were compared to the native ones established in a previous study.^14^

Theoretical mesh size of all mucus samples was calculated based on storage modulus (eq 1):

$$\xi ={\left(\frac{k_{B}\times T}{G^{\prime }}\right)}^{1/3}$$ 1

where *k*_B_ is the Boltzmann constant (1.38 × 10^–23^ m^2^ kg s^–2^ K^–1^), *T* is the absolute temperature in Kelvin, and *G*′ is the storage modulus. This equation estimated the mesh size assuming a cubic lattice.^19,20^

### Dry Weight and Water Content Determination

Approximately 200 mg of PNCM and PACM each was weighed on tared vials, sealed with parafilm perforated with a few holes, and frozen at −20 °C overnight. Freeze-drying was carried out for 48h using a Flexi-Dry MP, FTS systems from CiAB, Sweden, at −80 °C. Water content was calculated from the weight difference.

### Zeta Potential

Zeta potential was measured at 37 °C with a Litesizer 500 (Anton Paar) using an Omega cuvette with the Smoluchowski approximation. Before each of the measurements, the Omega cuvette was tested with the Applied Microspheres (Anton Paar) as a control. Samples were prepared as a dispersion of mucus 0.05–0.07% (w/v) in Milli-Q water. Each sample was measured five times following the assay guidelines developed in collaboration with Anton Paar, and three replicates were performed.

### Cryo-Scanning Electron Microscopy and Image Analysis

PNCM samples were kept on ice without freezing (to prevent ice crystal formation) during transport to the microscopy facility and then submerged in liquid nitrogen (−200 °C) with a Leica EM ICE high-pressure freezer (Leica, Wetzlar, Germany) at 325 ms and 2200 bar. Samples were placed in 200-μm deep gold-plated planchets with flat planchet tops. Prior to imaging, the flat top planchet was removed. PACM samples were prepared 1 day before high-pressure freezing and handled in the same manner as above.

Samples with planchets were stored in liquid nitrogen until imaging. At the beginning of the imaging session, samples were sublimated inside an Aquilos 2 cryo-FIB-SEM for 17 min at −110 °C and 5 × 10^–7^ mbar. Following sublimation, the Aquilos 2 was cooled to −190 °C, and the samples were sputter-coated with platinum to prevent charging. SEM images were obtained using an Aquilos 2 cryo-FIB-SEM instrument (Thermo Fisher Scientific). Images were collected using an Everhart–Thornley secondary electron detector with 3 kV, 13 pA, and a 1 μs dwell time. These settings were chosen to reduce the sample damage.

Images were analyzed with FIJI software by using the image binarization process. This process involved pixel thresholding (0–75 fixed values, the same for all images) to distinguish pores and mesh. Scale of measurements was set based on a micrograph scalebar. The “analyze particles option” was chosen, and the size range was set at minimum 0.1 nm (to eliminate noise) to infinity. Default circularity was chosen (between 0 and 1). Feret maximum and minimum diameters were obtained for comparison with the theoretical mesh size obtained from the storage modulus. The Feret measurement was also performed manually to compare with the binarization.

### Fluorescence Recovery after Photobleaching

Fluorescence recovery after photobleaching (FRAP) experiment was carried out, as described previously.^21^ Mucus samples (300 μg) were prepared with 3.75 μL of 10 mg/mL FITC-dextran stocks to achieve a final concentration of 0.125 mg/mL in the mucus. The FITC-dextran-loaded mucus samples were put onto a glass slide with a stacked coverslip setup to ensure a sample thickness of 100 μm. Experiments were conducted in a 37 °C temperature chamber. Images were recorded with a confocal microscope Zeiss CLSM 780 NLO, and data were collected using ZEN Black software (Carl Zeiss GmbH, Jena, Germany). At the beginning of experiments, samples were photobleached with a maximum laser power for 3 s. Bleaching areas and control areas with circular shapes (20 μm diameter) were made inside the rectangle-shaped frame to increase the speed of data acquisition.

Diffusivity values were calculated with the FRAPanalyzer^22^ by using double normalization settings:

$$I_{\mathrm{norm}}(t)=\frac{I_{\mathrm{ref}\text{\_}\mathrm{pre}}}{I_{\mathrm{ref}}(t)-I_{\mathrm{back}}(t)}.\frac{I_{\mathrm{frap}}(t)-I_{\mathrm{back}}(t)}{I_{\mathrm{frap}\text{\_}\mathrm{pre}}}$$ 2

and a diffusion model for the circular spot, based on modified Bessel functions:

$$\mathrm{FRAP}(t)=a_{0}+a_{1}.e^{-\tau /2(t-t\;\mathrm{bleach})}\cdot (I_{0}(\frac{\tau }{2(t-t\;\mathrm{bleach})})+I_{1}(\frac{\tau }{2(t-t\;\mathrm{bleach})}))$$ 3

$$\tau =\frac{w^{2}}{D}$$ 4

### Particle Tracking

Nanoparticle movement was investigated using a custom-built spinning disk confocal microscope based on an Eclipse Ti2 body (Nikon) using a 100×/1.42 NA Plan Apo Lambda objective (Nikon). Latex beads were studied in triplicate for each setting: polystyrene particles with sizes of 0.1, 0.2, 0.5, and 1 μm (carboxylate-modified and hence, negatively charged) and 0.1, 0.2, and 1 μm (amine modified and hence, positively charged) were studied in PACM PAA, PACM HEC, and PNCM. The suspended latex beads were sonicated following the manufacturer’s instructions, and thereafter, 0.3 μL of the particle suspension was mixed with 40 mg of mucus. Dilution volume was minimal (less than 1% of the mucus) to avoid influencing mucus viscosity. Each time-lapse video would be at least 100s in length with images acquired at 1 Hz in triplicate. Samples were incubated in a humidified chamber at 37 °C. To avoid thermal drift, samples in 18-well μ-Slides (Ibidi, Gräfelfing, Germany) were equilibrated for approximately 45 min before recordings were taken.^23^

Time-lapse movies were analyzed with the FIJI^24^ plugin MosaicSuite^25^ with particle tracker.^25^ For each type of particle, radius settings (in pixel units) were optimized to ensure particle detection. The cutoff and percentile were also set for determining stringency and signal intensity detection, respectively. Following the particle tracking process, images were obtained and trajectory files were saved, which were then analyzed with MPTHub.^26^ Trajectories of at least 100 particles were analyzed for each group of experiments. Following the image acquisition, we used the following input: a lag time (Δt) of 1000 ms, a width of the full frame of 182.83 μm, and a pixel size of 0.114 μm.

Mean-squared displacement (MSD) values ⟨Δ*r* ^2^(τ)⟩ were determined by

$$\left\langle {\Delta r}^{2}\left(\tau \right)\right\rangle ={\left[x\left(t+\tau \right)-x\left(t\right)\right]}^{2}+{\left[y\left(t+\tau \right)-y\left(t\right)\right]}^{2}$$ 5

where *x* and *y* denote the coordinates between consecutive τ intervals.^26^

Derivation of particle diffusivity from the calculated MSD value was obtained through

$$D_{\mathrm{eff}}=\frac{\left\langle \Delta r^{2}(\tau )\right\rangle }{2n\tau }$$ 6

$$D_{w}=\frac{kBT}{6\pi \eta r}$$ 7

$$\langle \Delta r^{2}(\tau )\rangle =4D_{0}\tau^{\alpha }$$ 8

where *n* refers to dimensionality, *D*_w_ is the theoretical diffusion coefficient of spherical particles in water, *kB* is the Boltzmann constant, *T* is the temperature, η is the fluid viscosity, *r* is the hydrodynamic radius of the particles, and *D*_0_ is the time-independent diffusion coefficient.^26^

Analysis times (ATs) were determined to be 100 and 10 s (10% of the whole video, as suggested by a previous study to reduce error^27^). Frame filters (FFs) were used to determine particle availability in a given number of frames to qualify them for analysis. Here, we selected 1FF, 10FF, 50FF, and 100FF, meaning only particles that were detected in 1, 10, 50, and 100 frames, respectively, were included in the analysis.

There is no general consensus regarding data analysis and filtering parameters to obtain a good fit of anomalous coefficient from a mean-squared displacement (MSD) log slope.^26,27^ Some studies report that increasing the time interval increases the error rate.^28^ For determining the anomalous coefficient (α), we chose 10% of the total time frame, corresponding to the MSD log slope (see Figure S1). To classify α into the ratio of transport mode, we refer to the suggestion by previous study:^26^ immobile (0.0 < α < 0.199), subdiffusive (0.2 < α < 0.899), diffusive (0.9 < α < 1.199), and active transport (1.2 < α < ∞).

### Microscale Thermophoresis

The interaction between FITC-dextran and three mucus samples (PACM PAA, PACM HEC, and PNCM) was studied by using a microscale thermophoresis (MST) fluorescent detector (Monolith NT.Automated) with capillary chips from NanoTemper Technologies GmbH (Germany). A stable temperature of 25 °C was maintained during the recording of the fluorescence levels of FITC-dextran and mucus samples at designated capillary positions. FITC-DEAE-dextran was used as the ligand after being dissolved in 10 mM MES buffer (pH 6.5) at a concentration of 2 μM based on a pretest. A pretest was performed to determine the detectable concentrations of FITC-dextran, using a Nano-Blue detector with a λ_ex_ of 495 nm and a λ_em_ of 519 nm (the recommended settings for FITC). The maximum concentration for each sample was determined through preliminary testing. Freshly prepared PACM PAA, PACM HEC, and PNCM were dispersed in 10 mM MES buffer as stock solutions (4, 25, and 25 mg/mL, respectively). The targets were prepared by serial dilutions of the mucus sample stock solutions with 10 mM MES buffer containing 0.3% Tween-20 (resulting final concentrations ranging from 0.0024 to 5 mg/mL) to prevent adsorption of the samples to the capillary chips. Mixtures of ligands and targets were inserted into the chips, followed by readings with 10% excitation power and low MST power for 3s pre-MST, 10s during MST, and 1s post-MST. All buffers and samples were degassed by sonication beforehand to avoid air bubble formation, and experiments were performed in triplicate.

The normalized fluorescence was plotted as a function of the concentration of the target to determine the dissociation constant (K_d_). The K_d_ indicates the equilibrium of the ligand–target complex and describes the concentration-dependent binding interaction. Reports and analyses of K_d_ fit were generated using MO.Affinity Analysis (NanoTemper) software.

### Statistical Analyses

Statistical analyses and graphs were produced using GraphPad Prism software 9.0. The ANOVA nonparametric test (Kruskal–Wallis test) was used for data assumed to have non-normal distribution. Wilcoxon paired *t-*tests were used for comparison of Feret diameters. Data cleaning (compiling values and converting units) was performed in Microsoft Excel. For the MST result, analysis and graph plotting were done with MO.Affinity Analysis (NanoTemper, Germany) software.

## Results and Discussion

### Physicochemical Characteristics of the Artificial Mucus Compared with the Native Colonic Mucus

In this study, we modified the polymer composition of the formulation described by Barmpatsalou et al.^14^ By doing so, we were able to produce an artificial mucus with greater similarity to PNCM in terms of pH, osmolality, and zeta potential.

At the initial stage of development, we tested PACM with pectin (from apple) as the backbone polymer (data not shown). However, the pectin induced phase separation, and therefore, this polymer was not explored further. For the other polymers, the optimal concentration of each gelling agent was determined by sequential addition and visual observation (Figure 1A), followed by rheological measurements.

**Figure 1:**
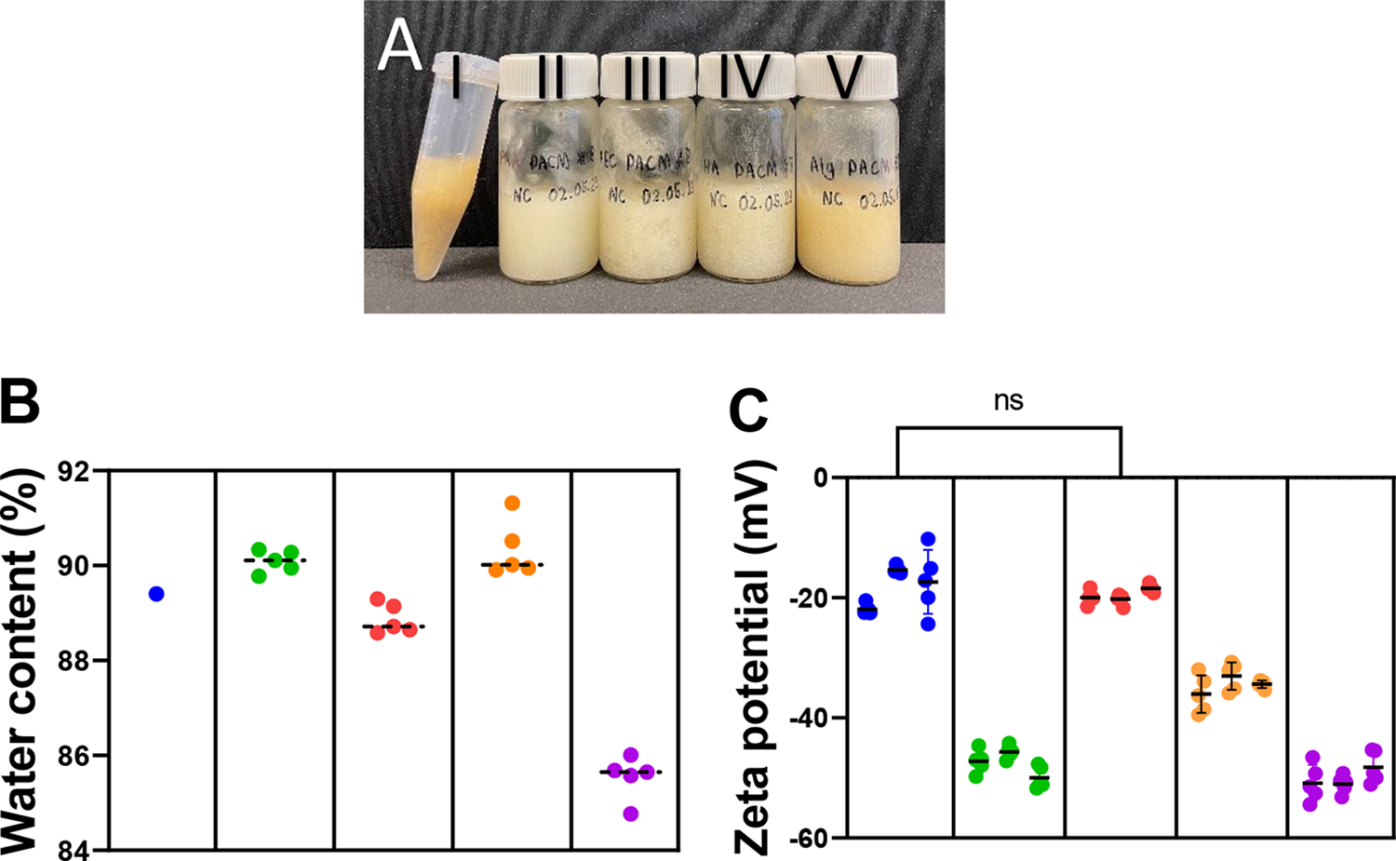
Mucus appearance and mucus physical characteristics. (A) Appearance of native mucus and the four porcine artificial colonic mucus (PACMs). (I) porcine native colonic mucus (PNCM); (II) PACM-poly­(acrylic acid), (III) PACM-hydroxyethyl cellulose, (IV) PACM-sodium hyaluronate; and (V) PACM-sodium alginate. PNCM and PACMs are similar in consistency and appearance. (B) Water content of PNCM and PACMs with different polymers: Green: poly­(acrylic acid) (PAA); red: hydroxyethyl cellulose (HEC); orange: sodium hyaluronic (SH); purple: sodium alginate (SA); blue: PNCM. Circles represent data points. Values of PNCM were taken from the literature.^14^ (C) Zeta potential of mucus samples dispersed in Milli-Q-water. Note the interindividual variability in PNCM (blue). Each sample was measured from three batches with five technical replicates.

In PNCM, the main gelling agent (mucins) constitutes around 2–5% (w/v) of the total volume of the formulation (Table 1). For the PACMs, the comparable volumes of the gelling agents were 1.5% (w/v) for PAA, 3% for SH and HEC, and 5% for SA. Initial gelling agent concentrations were determined from visual observation and literature study, followed by rheological measurement (Figure 2).

**Figure 2:**
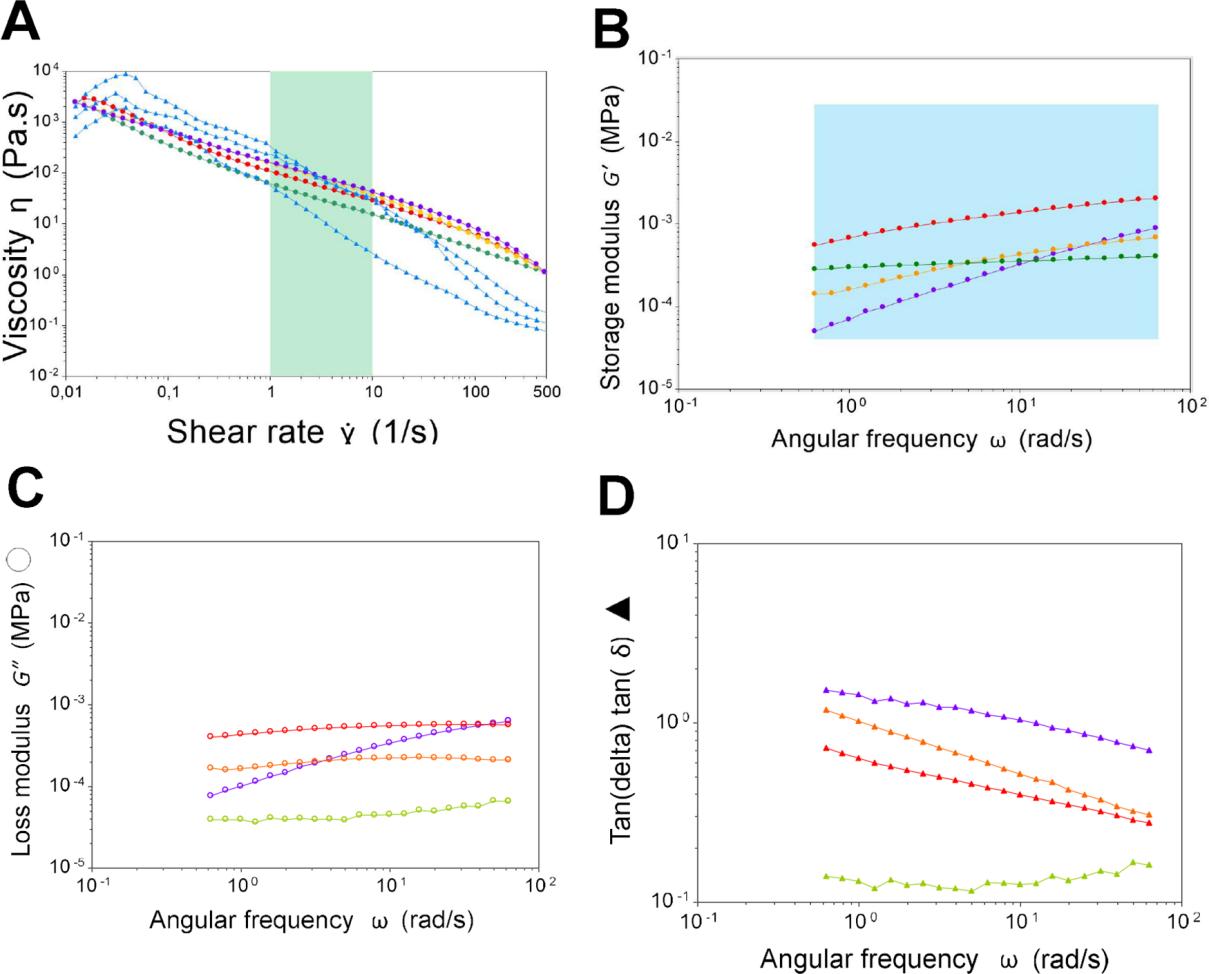
(A) Apparent viscosity vs shear rate and (B) storage modulus in the linear viscoelastic region (0.5% strain) of three porcine artificial colonic mucus (PACM) systems and native pig colonic mucus (PNCM, blue shadowed area); the corresponding loss modulus (C) and tan­(delta)­tan (D) in the linear viscoelastic region (0.5% strain). Green shadowed area indicates the physiological relevant shear rate of pig small intestine based on the literature.^14^ Values obtained in human colon based on MRI is within a similar range (3–10 1/s).^31^ Blue is PNCM, green is PACM PAA, red is PACM HEC, orange is PACM SH, and purple is PACM SA.

Barmpatsalou et al.^14^ reported the water content of the proximal region of porcine colon to be 89.4 ± 1.0%.^14^ The water content of a hydrogel, such as mucus, is critical because it influences the diffusivity of molecules and particles within it.^29^ Of the systems explored herein, PACM PAA, PACM HEC, and PACM SH closely mimicked the PNCM water content, while PACM SA had significantly lower water content at the concentrations providing good hydrogels (Figure 1B).

An important factor impacting the diffusion of molecules and particles is the zeta potential. It is a macroscopic measure of surface charge of the hydrogel and—depending on the charge of the molecules and particles diffusing in the hydrogel—causes interactions and potential binding through electrostatic interactions.^30^ Therefore, any artificial mucus should mimic the PNCM zeta potential as closely as possible. The mean value for PNCM was −18.2 (±4.0), while the values for PACMs were −47.6 (±2.4) for PACM PAA, −20.9 (±1.4) for PACM HEC, −34.5 (±2.5) for PACM SH, and −50.0 (±2.7) for PACM SA. For the four polymers investigated, the HEC system performed the best, with no statistically significant difference to PNCM zeta potential (Figure 1C).

### Macrorheological Properties of PNCM and PACM

Macrorheological properties, such as viscosity sweep profile, storage modulus in linear viscoelastic region (LVR), and elasticity behavior, were also evaluated in the PACM models (Figures 2 and S4). Similar to PNCM, all PACMs showed shear-thinning behavior, in which the viscosity values would decrease upon exposure to increasing shear rates (Figure 2A). Mucus is a hydrogel and hence will have the strength to withstand shear stress to a certain level. The region in which the mucus has the capacity to withstand stress before deformation is defined as LVR (Figure S4). With the strain determined from the LVR, we could perform a storage modulus measurement (Figure 2B) and achieve a comparison while validating that the gel structural integrity was still present.

A higher storage modulus than a loss modulus indicates viscoelastic solids, with structures of strong interaction forces. In contrast, a higher loss modulus than storage modulus indicates viscoelastic liquids, which is the characteristic of non-cross-linked polymers.^32^ Only the rheological behavior of the PACM SA model differed from that of PNCM, indicating that PACM SA behaves as viscoelastic liquids (Figure S4). While PNCM, PACM PAA, PACM HEC, and PACM SH showed characteristic of viscoelastic solids (Figure S4), they still differed from PNCM in crack point (crossover between storage modulus and loss modulus). This indicates that they would have different structural strengths to resist strain. However, the storage modulus and viscosity in physiological shear rate range (1–10 s^–1^) of all PACM samples fall in the range of PNCM.

### Optimization of PACM HEC

As PACM HEC mimicked the native colonic mucus best in the above assays, we chose to continue to work with it as the polymer backbone. However, pH may influence the viscosity of the mucus, as well as the charge of any molecule present in the mucus layer, thus affecting drug diffusivity.^34^ We therefore studied the HEC-based hydrogel with another buffer system at a pH closer to that of the physiological one. MES buffer was replaced with BES buffer, which fitted the pH of PACM HEC into the range of the native mucus (Table 1). This change did not affect the storage modulus (see Figure S3). In further optimization, we reduced the HEC from 5 to 3% (w/v) to provide values accommodating the middle range of storage modulus value (Figure S2). This resulted in a storage modulus of PACM HEC closer to the PNCM average, herein, determined from samples from six pigs (Figure S1).

### Microstructure of PACM Compared with PNCM

The porous structure of the mucus network was investigated with Cryo-SEM (Figure 3), and images were used to determine the pore size of the mucus. In the native mucus sample, the Feret maximum and minimum diameters were different from each other (Figure 4A), and hence, the pores were not spherical.^35^ In a previous study, the pore diameter was determined based on the smallest diameter of the void space enclosed by the pore walls, which agrees with the mesh radius concept.^36^ We therefore compared the Feret minimum diameter of the artificial mucus with the same measured in the native one. Calculation of pore size based on the storage modulus (Figure 4B,C) was performed from the rheological characterization (Figure 2B) based on eq 1 and would be referred to as the theoretical mesh size. Following this step, the Feret minimum diameter from the analyzed images was compared with the theoretical mesh size (Figure 4B). The binarization thresholding is critical in the analysis of the images. Manual measurement resulted in significantly (tested by the Kruskal–Wallis method) larger pore size (median of 68 nm) as compared to the methods by binarization (median of 25 nm) and theoretical mesh size (median of 13 nm). All three methods resulted in a relatively wide range of mesh size. The theoretically calculated pore size from the storage modulus ranged from 5 to 55 nm, the manual pore size from 20 to 312 nm, and the binarization from 8 to 1181 nm. Hence, the range derived from the theoretical calculation was smaller and less variable but still in the range of the Feret min diameter of the Cryo-SEM binarization values.

**Figure 3:**
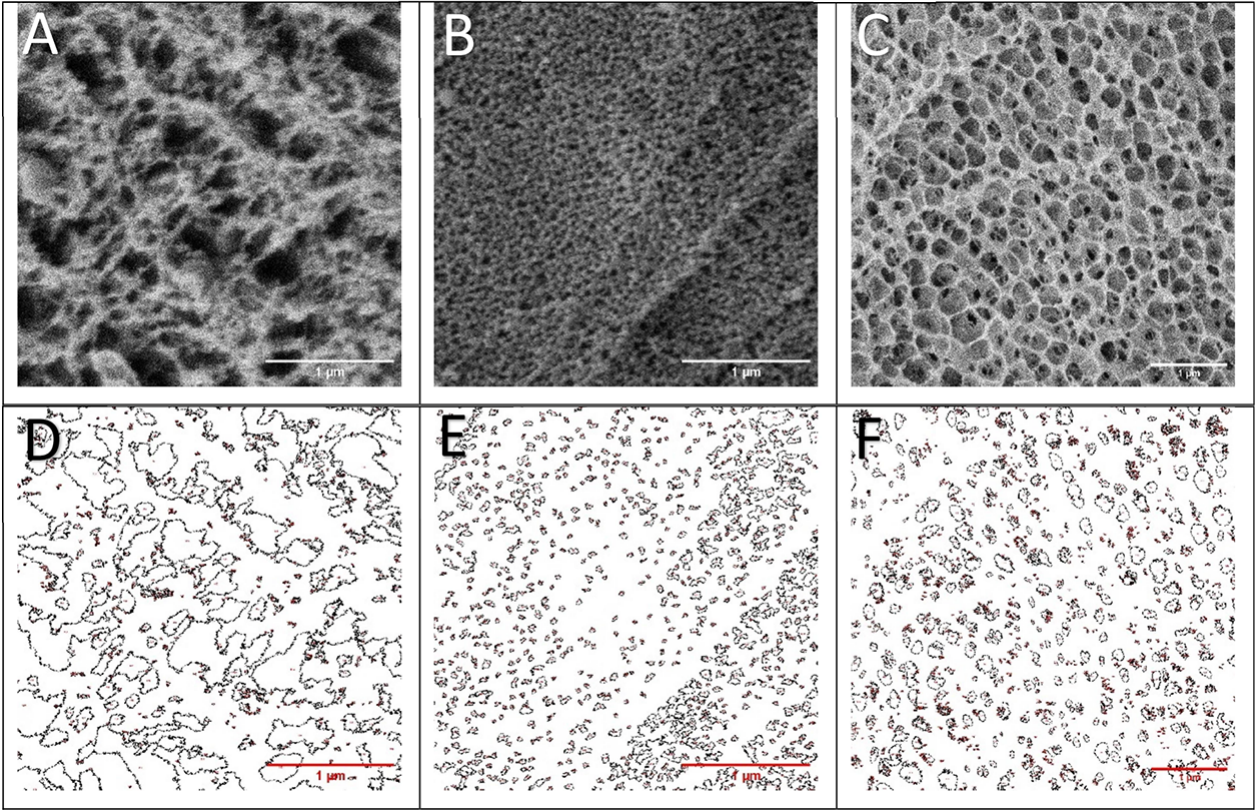
Representative Cryo-SEM micrographs of native mucus (A, D), PACM PAA (B, E), and PACM HEC (C, F). Resulting images from a binarization process allow detection of the pores (D–F).

**Figure 4:**
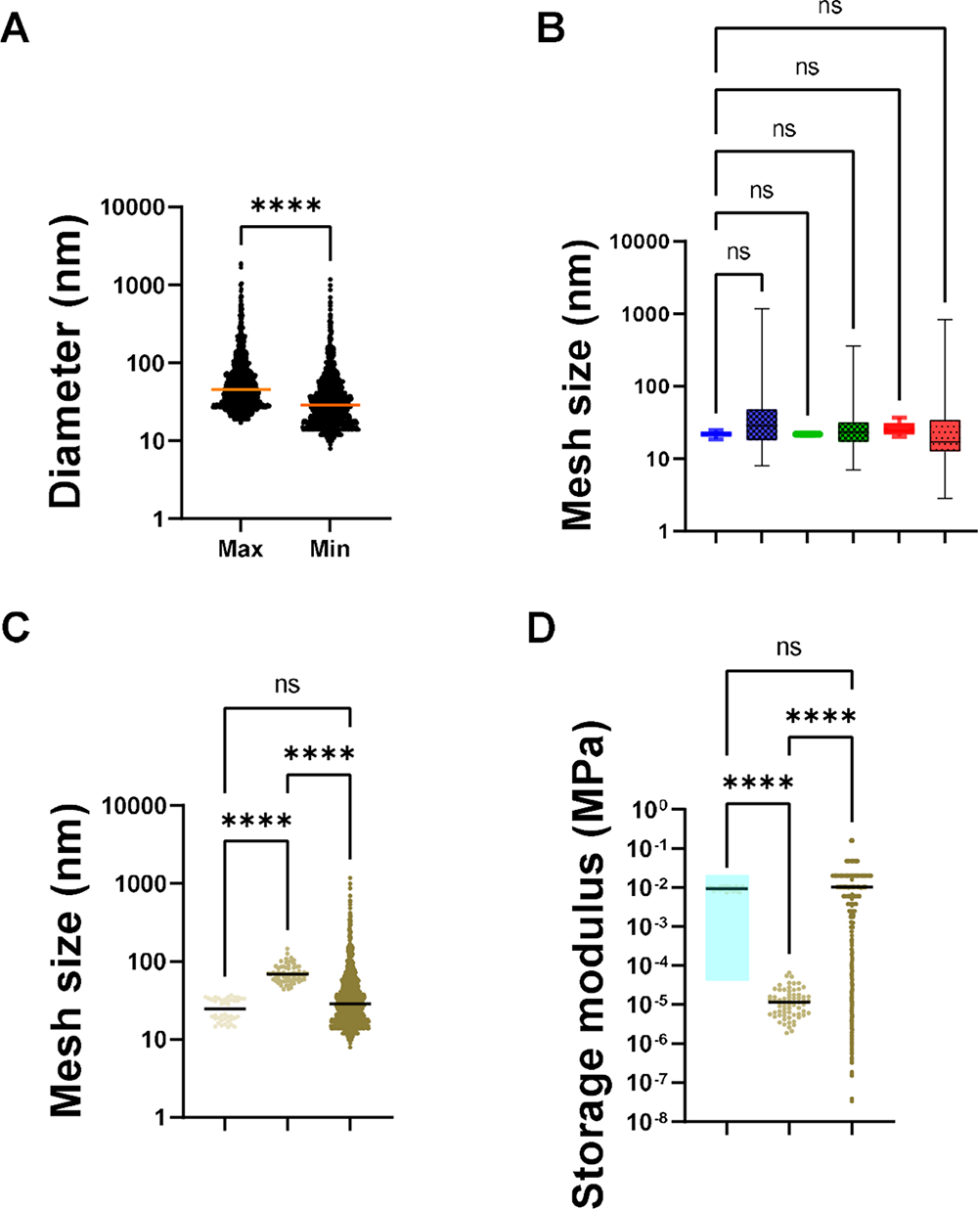
Pore and mesh size. (A) Feret maximum and minimum diameters of pores in porcine native colonic mucus. Wilcoxon paired *t-*test was performed to assay for statistically significant difference. A significant difference in max and mean diameter was observed (****=*p* < 0.0001). (B) Comparison of theoretical mesh size from storage modulus measurement vs observed Feret min diameter from the Cryo-SEM image for PNCM, PACM PAA, and PACM HEC. Statistical test was performed with the Kruskal–Wallis ANOVA test. Framed square indicates values from thresholded Cryo-SEM images, while nonframed square indicates theoretical values. Blue: PNCM; green: PACM PAA; red: PACM HEC. (C) Comparison of Feret minimum diameter in a PNCM micrograph, as measured by manual Feret approximation vs binarization pixel thresholding. See also Figure S1 for comparison with the theoretical mesh size derived from the storage modulus value. Light brown: Calculated from storage modulus; brown: Cryo-SEM manual; dark brown: Cryo-SEM thresholding. (D) Storage modules calculated from the determined mesh size. Eq 1 was used to determine if the storage modulus value could be estimated from the mesh size. Light brown: Storage modulus measured from the rheometer; brown: storage modulus calculated from mesh size based on manual; dark brown: storage modulus calculated from mesh size based on thresholding.

The Feret minimum diameter and theoretical mesh size of PNCM were similar to each other (Figure 4C). Furthermore, we identified that the freezing protocol significantly affected sample preparation and, hence, the resulting pore size. As described by Efthymiou et al.,^37^ high-pressure freezing is crucial for maintaining the amorphous state of liquid content to prevent it from crystallizing. Crystallization may expand the pores of the hydrogel, leading to inaccuracy in the pore size measurement.^37^ After adapting the freezing protocol by using a high-pressure freezer, the pore sizes in our study were significantly smaller than those reported in previous studies.^11,14^ We believe our measurements provide a more realistic image of the network structure since the risk of ice crystal formation is significantly reduced.

The standard protocol to measure the storage modulus of hydrogels uses bulk rheology. However, the choice of geometry and setup influences the results and the accuracy.^38^ We therefore chose image binarization using Cryo-SEM micrographs to cross-validate the pore size data calculated with rheology (Figure 4D). After validating the binarization method for image processing of PNCM, it was used to determine the pore size of the PACMs. We compared the theoretical (calculated from the storage modulus value using eq 1) and Cryo-SEM thresholds (calculated by binarization of Cryo-SEM images). We concluded that the two different methods produced very similar results (Figure 4B).

The HEC pore size was determined to be nonstatistically significantly different from that observed in PNCM, and the values were still in the same range as those of the native mucus. The median values calculated from the Cryo-SEM images were: 32.47 nm for PACM HEC, 26.17 nm for PACM PAA, and 47.77 nm for PNCM. Hence, in general, the mesh pore size was similar in the artificial mucus models and the native mucus. In a previous study, the pore size of pig jejunum mucus was around 200 nm.^39^ As it has lower viscosity than mucus from the pig colon—and since viscosity and solid contents correlate negatively with pore size^20^—a pore size of <200 nm is expected. Human airway mucus, which, in some situations, can be more viscous than small intestinal mucus,^40,41^ is also reported to have pores <100 nm.^42^

The values were similar for those from the storage modulus (taken by direct measurement with a rheometer, Figure 2) and the equation-based mesh sizes (Figure 4D). Hydrogel mesh size (ξ) is defined as the linear distance between two adjacent cross-links,^43^ while pore size is defined as the diameter (Feret’s max and min diameter) that fits within a specific pore.^35^ Taken together, both mesh size and pore size (from experimental and theoretical calculations with cryo-SEM and rheometer methods) values seem to fit the current knowledge of mucus pore sizes, with the PACM able to mimic PNCM pore size and distribution.

### Particle Diffusivity in Selected Artificial Mucus Models

Diffusivity in the mucus layer is an important indicator of drug penetration and absorption. It is affected by the factors described above (viscosity, pH, zeta potential, binding to components such as lipids, mucin, albumin, and, in artificial mucus, the selected polymer). In a previous study, we investigated diffusion of macromolecules in PACM PAA and PNCM.^11^ In the current study, we therefore focused on studies of nanoparticles after establishing that PACM HEC had similar diffusivity values as PACM PAA for the FITC-dextran as the model of macromolecules (Figure S5). Model nanoparticles with different charges (anionic and cationic) and sizes (0.1–1 μm) were used to simulate diffusivity in mucus of undissolved drug particles or nanosized drug delivery systems. The data were then collected with particle tracking analysis. During the analysis, the choice of frame filter is critical since it may introduce bias if more immobile particles are included or actively diffusing particles are excluded. Furthermore, a too narrow filter may overestimate diffusion, whereas a too broad one may introduce larger errors.^27^ After exploring the impact of the analysis time on the data (Figure S7), 10 s was chosen, which corresponded to 10% of the total video recording. A frame filter (FF) of 10 was chosen, i.e., particles present in 10 frames of the recording, corresponding to 10% of analysis time (AT), were included.

Mean-squared displacement (MSD) quantifies the deviation of the particle position from the initial to the final time. The average MSD in PACM HEC ranged from 1.9 × 10^–3^ to 1.8 × 10^–1^ μm^2^ (Figure 5E). A slightly smaller range was observed in the PACM PAA (7.2 × 10^–3^–1.2 × 10^–1^ μm^2^; Figure 5C), whereas a larger range, indicating greater flexibility and variability in the network mesh, was observed in native colonic mucus (7.0 × 10^–2^–1.4 μm^2^; Figure 5A). The corresponding *D*_eff_ can be derived in line with the MSD value (Figure 5B,D,F) by using eq 6. A similar analysis has been made in pig jejunum mucus, which reports *D*_eff_ values around 0.1 μm^2^/s.^39^ Considering the higher viscosity of PNCM^14^ (compared to jejunum mucus), it is logical to observe lower values in the native and artificial colonic mucus. However, the mean *D*_eff_ of PACM PAA (2.7 × 10^–3^) and PACM HEC (2.7 × 10^–3^) was lower range of average *D*_eff_ values compared to PNCM (2.6 × 10^–2^) in 10s AT and 10FF.

**Figure 5:**
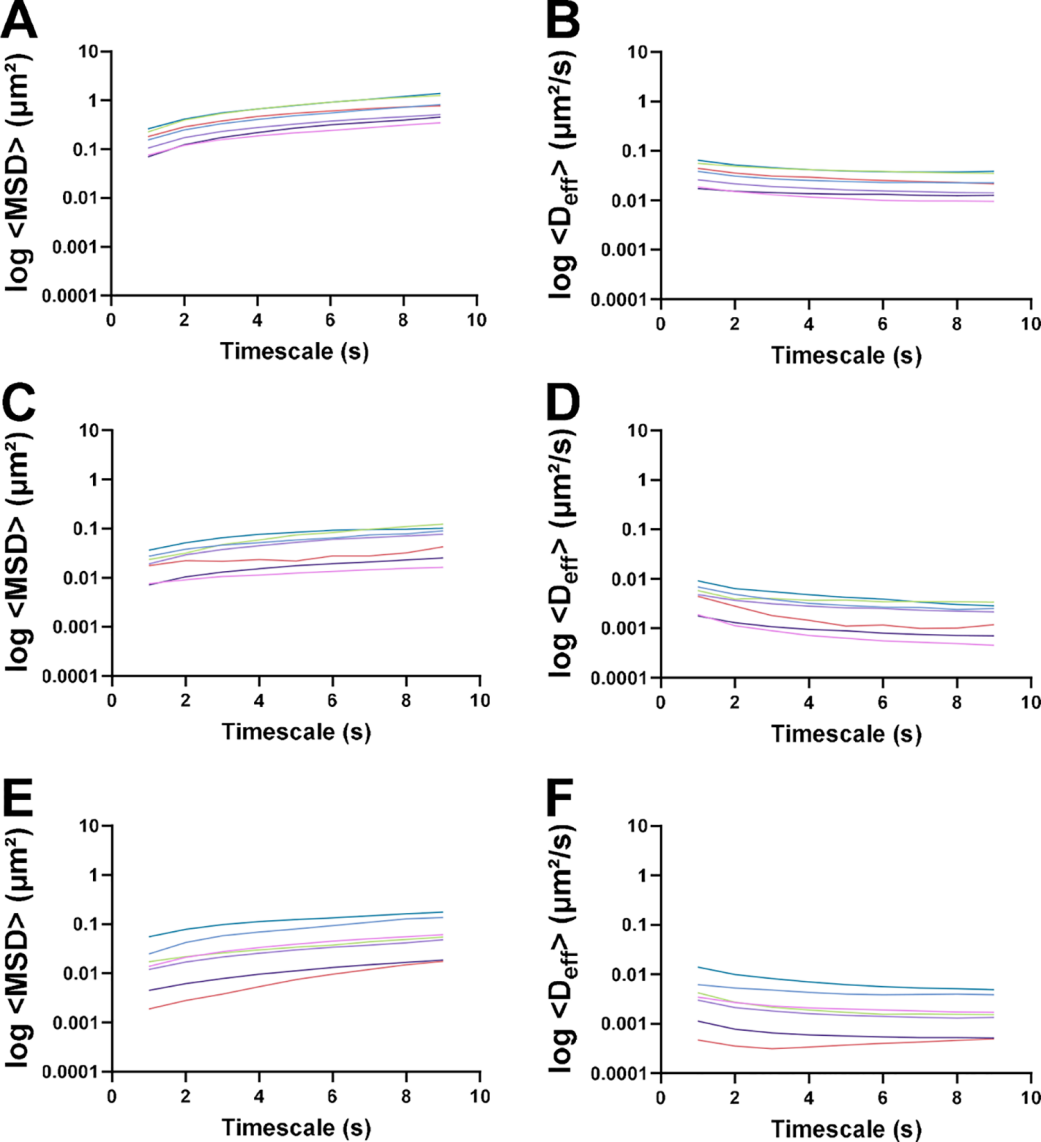
Mean-squared displacement (MSD) range and effective diffusion (*D*_eff_) are measured by particle tracking. Analysis time was 10s with ten frame filters for polystyrene nanoparticles of different charges. Sizes studied were 0.1, 0.2, 0.5, and 1.0 μm anionic nanoparticles, and 0.1, 0.2, and 1.0 μm cationic nanoparticles. The following matrices were investigated: porcine native colonic mucus (A, B), poly­(acrylic acid) artificial mucus (C, D), and hydroxyethyl cellulose (E, F). The effective diffusion of nanoparticles was calculated from the MSD and is shown in eq 6. Light blue: 0.1 μm (−); light purple: 0.2 μm (−); pink: 0.5 μm (−); green: 1 μm (−); teal blue: 0.1 μm (+); dark blue: 0.2 μm (+); red: 1 μm (+).

The particle tracking analysis indicated that the 1 μm particles (both negatively and positively charged) diffused faster in the mucus than the smaller sizes, which may seem counterintuitive. However, this phenomenon is in line with other experimental and simulated data.^30,44^ Smaller (high surface charge density) particles diffuse more slowly than the larger ones (less surface charge density) due to attractive interactions between the hydrogel backbone (mucus in this case) and the nanoparticles.^44^ The charged nanoparticles (both cationic and anionic nanoparticles) diffuse slower than the uncharged ones^45^ due to constant repulsion and attraction forces from components in the mucus (e.g., mucin).^44^ In addition, the larger particles are still mobile in the mucus because the network matrix has a broad pore size distribution (Figure 4; up to 1200 nm for PNCM, 360 nm for PACM PAA, and 830 nm for PACM HEC), even if the nanoparticle size is larger than the average mesh size.^44^

### Transport Mode and Binding to Native and Artificial Mucus

The slope of the mean square displacement (MSD) value is called the anomalous coefficient (α), which can be derived from eq 8. Eq 8 also explains the importance of selecting the analysis time (AT) (Figure S7). This value of each particle trajectory plot was calculated from the MSD curve. Data were excluded for particles classified as active transport (α > 1.199; Figure S9). The α value (Figure S8) corresponds to the dominant type of movements and is sorted into four classes: immobile, subdiffusive, diffusive, and active transport. Most of the negatively charged nanoparticles were transported in a subdiffusive way (0.2< α < 0.899; Figure 6). This is in accordance with the findings by Schuster and colleagues, who sampled mucus from the human respiratory tract. They reported MSD α-values <1 for the negatively charged polystyrene nanoparticles, indicating that subdiffusive behavior dominates.^42^

**Figure 6:**
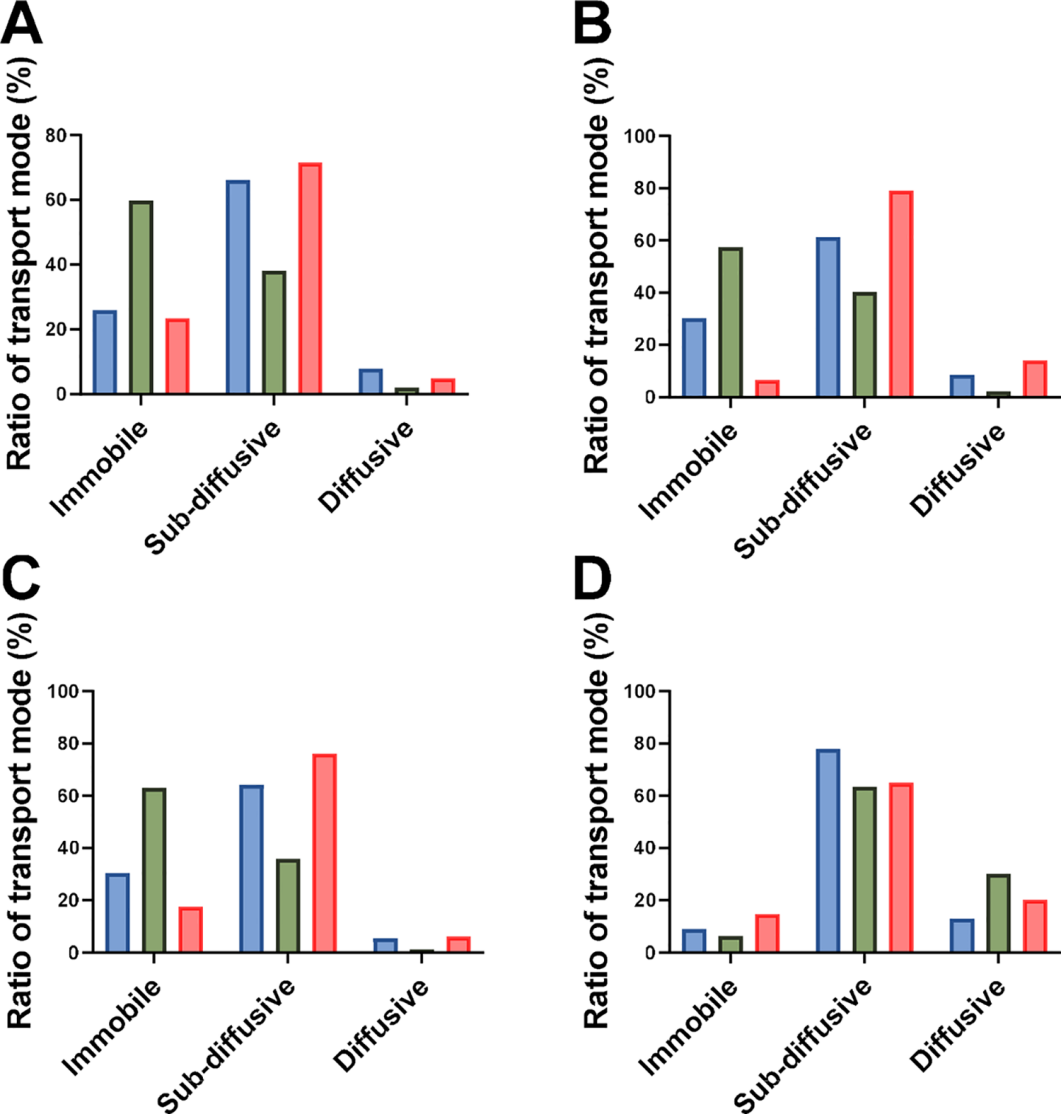
Ratio in % of transport mode (immobile, subdiffusive, and diffusive) for anionic nanoparticles with different sizes: (A) 0.1 μm, (B) 0.2 μm, (C) 0.5 μm, and (D) 1.0 μm. Color code indicates the mucus types: blue for PNCM, green for PACM PAA, and red for PACM HEC.

More of the positively charged (amine-modified) polystyrene nanoparticles were in the immobile state, most probably due to binding interactions (Figure 7). That cationic nanoparticles tend to be more immobilized have been observed previously. For example, positively charged nanoparticles diffuse 20–30 times slower than negatively charged ones in porcine jejunal mucus.^10^ Surprisingly, the 1 μm nanoparticles in PACM HEC were transported predominantly by diffusion (Figure 7C). We therefore followed up on these findings with additional measurements of their hydrodynamic sizes and zeta potential (Table S2). It was shown that they had a zeta potential closer to neutral, and the standard deviation of the hydrodynamic sizes was high. This indicates aggregation, which may further influence the anomalous behavior observed (Figure 7C). Aggregation can be induced by the addition of high concentrations of polystyrene nanoparticles, which, in turn, may collapse mucus fibers into bundles^46^ and further contribute to anomalous diffusion behavior.

**Figure 7:**
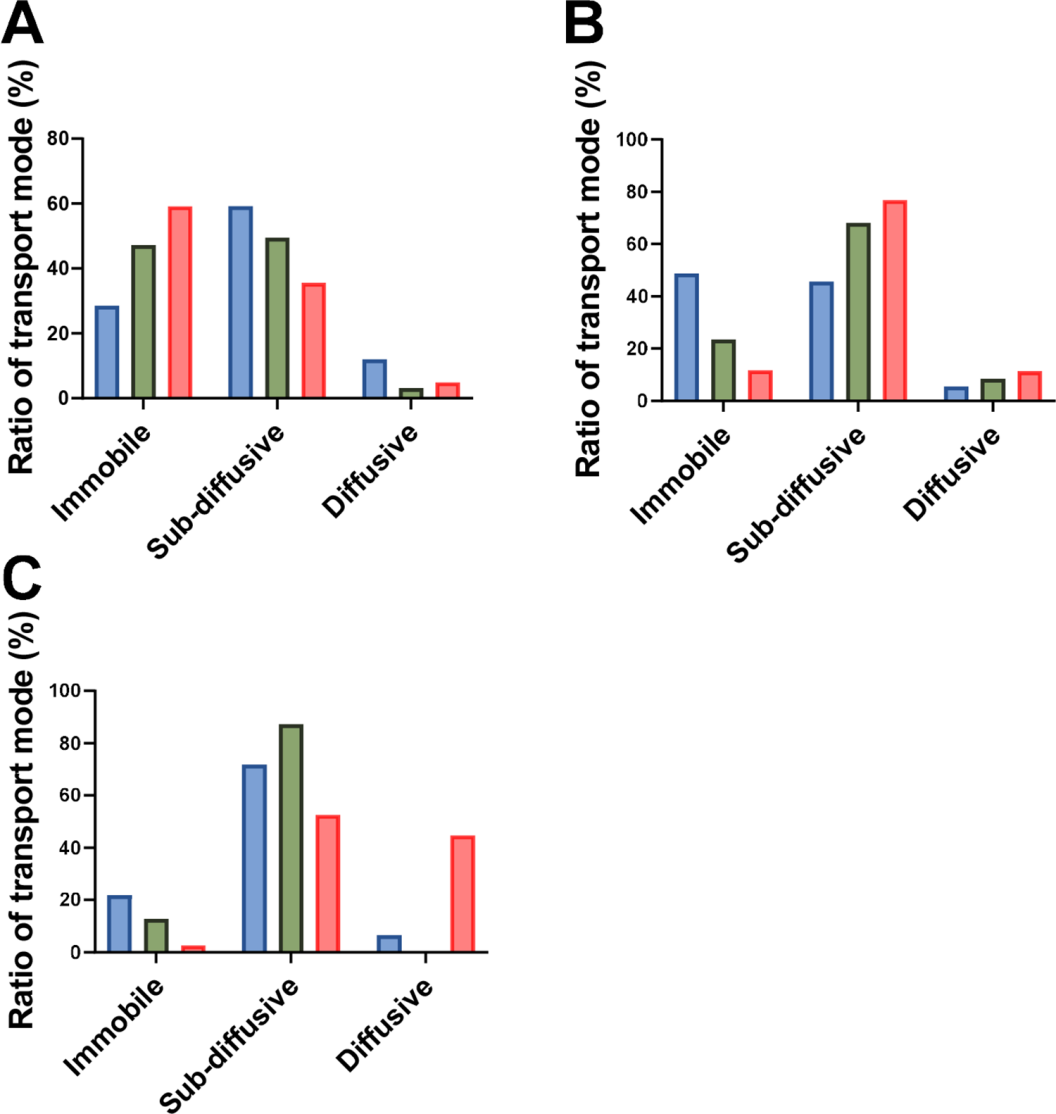
Ratio in % of transport mode (immobile, subdiffusive, and diffusive) for cationic nanoparticles with different sizes: (A) 0.1 μm, (B) 0.2 μm, and (C) 1.0 μm. Color code indicates the mucus types: blue for PNCM, green for PACM PAA, and red for PACM HEC.

The data presented in Figures 6 and 7 were used to identify which of the PACMs most closely mimicked the transport mode of the nanoparticles in the PNCM (Table S3). Currently, there is no clear consensus on how to compare transport modes and filter the data from particle track experiments.^26^ However, nanoparticles with α > 1 (active transport) have been excluded in previous studies;^47^ hence, we focused on the subdiffusive transport mode (0.199 < α < 0.899; Figures S7 and S8). On this basis, we concluded that PACM HEC was more similar to PNCM than PACM PAA (Table 2).

**Table 2:** Final Optimized Models of Two PACMs Compared with PNCM

sample	[optimal] of gelling agent (w/v)	pH (*x̅* ± SD)	osmolality (mOsm/kg) (*x̅* ± SD)	water content (*x̅* ± SD) (%)	zeta potential (mV)	storage modulus in LVR (MPa)	viscoelastic gel-like behavior	pore size (theoretical) (nm)	pore size (binarization) (nm)	FRAP diffusivity correlation	range of ⟨ MSD⟩ (μm^2^)	range of ⟨*D*_eff_⟩ (μm^2^/s)	similarity to PNCM: transport mode based on α value classification and binding profile by MST
PNCM	2–5% mucin^14^	7.3–7.5 ^14^	N/A	89.4 ± 1.0 ^14^	–15 to −22	4.5 × 10^–5^ to 2.6 × 10^–2^	yes	5.21–55.44	8.00–1181.00	[benchmark]	7.0 × 10^–2^ to 1.4	9.6 × 10^–3^ to 6.5 × 10^–2^	[benchmark]
PACM in MES buffer	1.5% PAA	7.14 ± 0.28	132 ± 1	90.1 ± 0.6	–45 to −55	2.8 × 10^–4^ to 4.1 × 10^–4^	yes	18.31–25.05	6.75–1041.00	similar	7.2 × 10^–3^ to 1.2 × 10^–1^	4.6 × 10^–4^ to 9.1 × 10^–3^	less similar
PACM in BES buffer	3% HEC	7.30 ± 0.07	134 ± 2	88.9 ± 0.7	–19 to −23	7.5 × 10^–5^ to 4.8 × 10^–4^	yes	19.80–40.20	2.82–827.90	similar	1.9 × 10^–3^ to 1.8 × 10^–1^	3.1 × 10^–4^ to 1.4 × 10^–2^	more similar

The majority of nanoparticles had subdiffusive transport whether in PNCM, PACM PAA, or PACM HEC. Thus, the artificial mucus replicated the size filtering and interaction filtering phenomena of the PNCM. There was a trend of increasing α as nanoparticle size increased (Table S4). This observation has also been reported in human cervical mucus by Cobarrubia et al. They showed that increases in the α of −COOH-modified polystyrene beads positively correlated with increasing size.^48^ It has also been shown that transport mode and movement for 0.1 and 0.2 μm nanoparticles in mucin solution are significantly different from those measured in native pig jejunal mucus.^10^ These differ even more as a result of the 10- to 100-fold higher viscosity in the colonic mucus compared to the jejunal.^14^ The *D*_w_/*D*_eff_ ratio gives an overview of theoretical diffusion in water per effective diffusion in the mucus (hydrogel matrix). Higher values mean more hindrance to diffusion and vice versa. A previous study reports a higher *D*_w_/*D*_eff_ ratio for 0.2 μm −COOH polystyrene beads than the 0.1 and 0.5 μm ones,^42^ which is in agreement with our findings for PACM HEC. Another study, with human chronic rhinosinusitis mucus, reports a *D*_w_/*D*_eff_ ratio of around 2300 for 0.2 μm −COOH polystyrene beads.^49^ This value is close to the ratio of 2349 for our PACM HEC. The relatively lower hindrance in the PNCM may be due to more water pockets there than in the homogeneous PACM. These pockets were seen in the video microscopy (data not shown).

Finally, we used microscale thermophoresis (MST) (Figure S11) to study binding between molecules and mucus.^21,50^ Cationic FITC-dextran 4KDa was chosen due to previously observed binding to mucus^21^ and observed hindered diffusivity, in contrast to the anionic and neutral FITC-dextran (Figure S5). Here, binding between the positively charged macromolecules and PACM HEC had a kinetic profile more similar to PNCM than that of PACM PAA (Figure 8). The optimization of the gel to produce a zeta potential reflective of the native porcine colonic mucus (Figure 1) is likely the reason for a better estimation of binding since PACM PAA is more negatively charged than either PACM HEC or PNCM (Figure 1).

**Figure 8:**
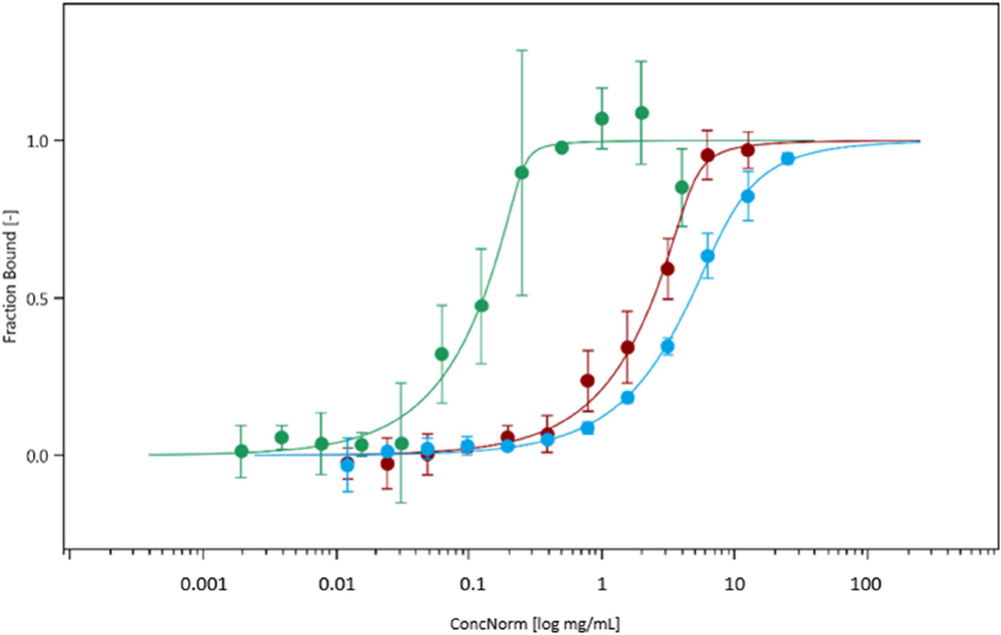
Binding profile between cationic FITC-dextran 4K and PACM PAA (green), PACM HEC (red), and PNCM (blue). Mucus samples were diluted to create a series of gradient concentration suspended in MES buffer.

## Conclusions

In developing artificial mucus, the polymer must be carefully selected if it is to mimic all of the different properties of the physiological mucus. Most studies use diffusion as the sole characteristic. In our work, we showed the impact of the gelling polymer on diffusion as well as on transport mode, movement patterns, and binding to the mesh. The HEC polymer resulted in zeta potential, binding, and transport mode similar to the native colonic mucus. Furthermore, many methods and techniques used herein show the importance of sample preparation and data analysis to correctly measure the pore size of the mesh. We present a new method for cross-validation of pore size determination by using two complementary techniques: Cryo-SEM and rheological characterization (storage modulus). We conclude that existing protocols for artificial mucus need to be revised with regard to both hydrogel gelling agent types and sample preparation. The protocols presented here, with respect to hydrogel composition, preparation, and characterization, generate biomimetic artificial mucus models that accurately inform on mucus influence on absorption of drugs, particles, and drug delivery systems.

## Supplementary Material



## Data Availability

Data is available upon request.

## Notes

Storage modulus of mucus samples, amplitude sweep showing linear viscoelastic region (LVR) of the mucus samples, diffusion coefficient from FRAP experiments, particle tracking workflow, particle tracking data analysis, capillary profiles and microscale thermophoresis experiments, artificial colonic mucus preparation protocol, and details of nanoparticle properties (PDF)
