# Supplementary material for: Optimized Artificial Colonic Mucus Enabling Physiologically Relevant Diffusion Studies of Drugs, Particles, and Delivery Systems
Source: Mol Pharm. 2025 Jun 10;22(7):4032–45. doi: 10.1021/acs.molpharmaceut.5c00298 (PMC12239078; doi:10.1021/acs.molpharmaceut.5c00298)
Supplement: Supplementary file 1 [file mp5c00298_si_001.pdf]

Supporting information

Optimized artificial colonic mucus enabling physiologically relevant diffusion studies of drugs, particles and delivery systems

Marco Tjakra[1,2], Nopdanai Chakrapeesirisuk[1], Magdalena Jacobson[4], Mikael E. Sellin[5,6], Jens Eriksson[5], Alexandra Teleki[2,3], Christel AS Bergström[1,2]

[1]Department of Pharmacy, Uppsala Biomedical Center, Uppsala University, 751 23 Uppsala, Sweden.

[2]The Swedish Drug Delivery Center, Department of Pharmacy, Uppsala University, Box 580, SE-751 6 23 Uppsala, Sweden

[3]Department of Pharmacy, Science for Life Laboratory, Uppsala University, 751 23 Uppsala, Sweden

[4]Department of Clinical Sciences, Faculty of Veterinary Medicine and Animal Science, Swedish University of Agricultural Sciences, Box 7054, SE-750 07, Uppsala, Sweden

[5]Department of Medical Biochemistry and Microbiology, Uppsala University, 751 23 Uppsala, Sweden

[6]Science for Life Laboratory, 751 23 Uppsala, Sweden

Content

Figure S1 Storage modulus area of PNCM 3
Figure S2 Storage modulus of PACM HEC modified by polymer amount 4
Figure S3 Storage modulus of PACM HEC-with two buffer systems 5
Figure S4 Amplitude sweep measurements of PACMs 6
Figure S5 Diffusion of fluorescently labelled dextrans in artificial mucus (PACM) 7
Figure S6 Workflow of video and trajectory data analyses 8
Figure S7 Average mean square displacement (MSD) for the three type of mucus 9
Figure S8 Anomalous coefficient distribution to classify particle movement 11
Figure S9 Anomalous coefficient distribution (only subdiffusive) 12
Figure S10 Capillary profiles of PACM and FITC-dextran interaction 13
Figure S11 Microscale thermophoresis of 4K cationic FITC-dextran 14
Table S1 PACM core formulation preparation protocol 15
Table S2 Hydrodynamic sizes and zeta potential of polystyrene nanoparticles 16
Table S3 Summary of differences between the two datasets 17
Table S4 Mucus systems with nanoparticle properties, average α, and diffusion in water (D0) 18

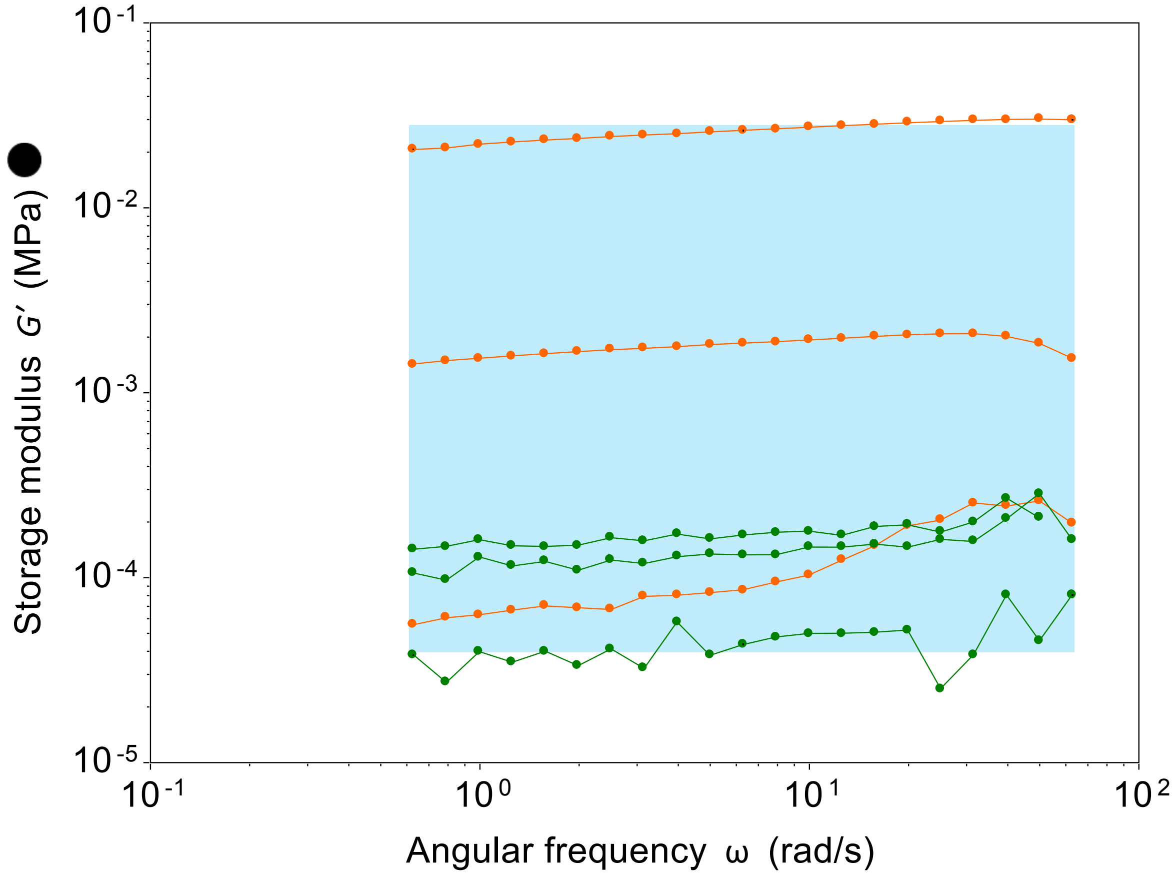

Figure S1. Storage modulus area of PNCM, based on data from previous publications. Orange line represents data from Barmpatsalou et al. 2021 and green line represents data from Barmpatsalou et al. 2023. Each line represents one replicate from one pig. Circles represents storage modulus data points. Light blue background corresponds to range of PNCM storage modulus between mean of the lowest (4,51e-5 MPa) and the highest (2,61e-2 MPa).

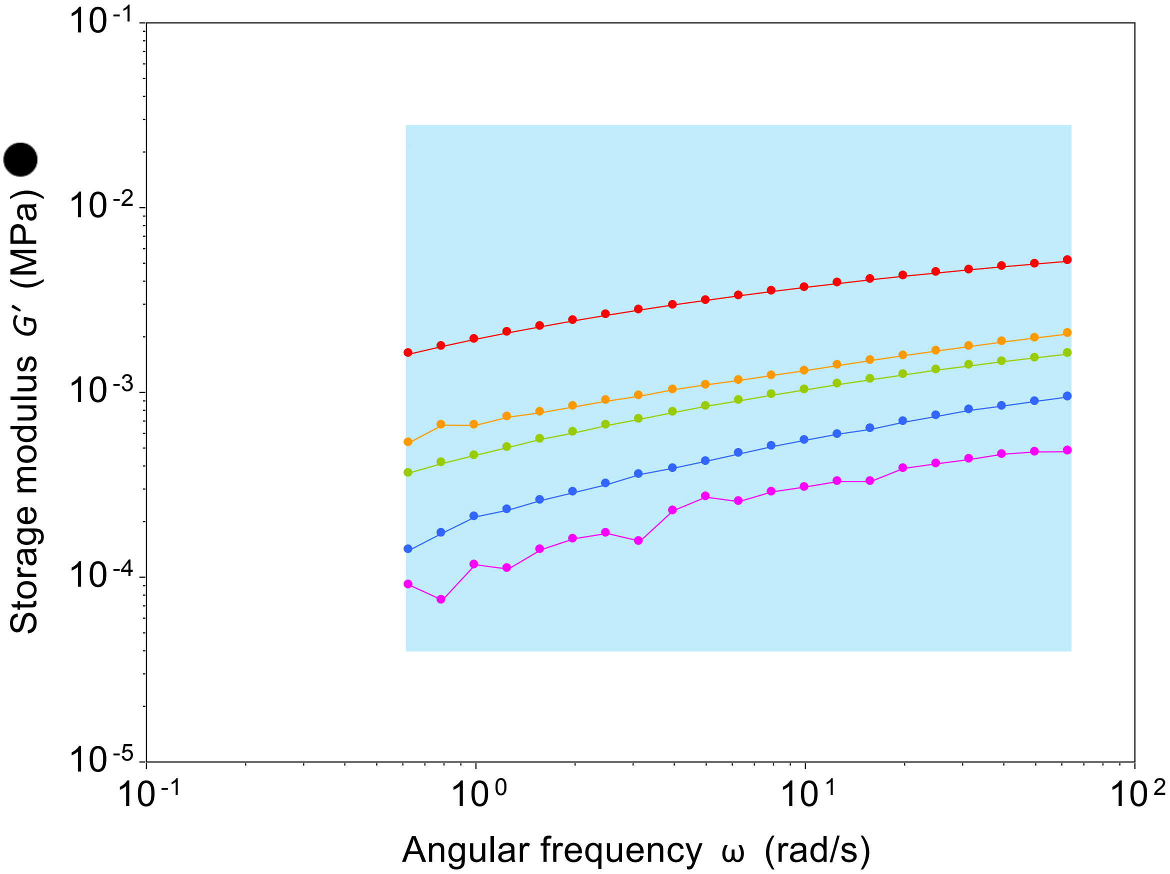

Figure S2. Storage modulus of PACM HEC modified by polymer amount. Different color corresponds to different amount of hydroxyethyl cellulose (HEC) polymer; Red: 600mg (6% w/v); Orange: 500mg (5% w/v); Green: 450mg (4.5% w/v); Blue: 400mg (4% w/v); Purple: 300 mg (3% w/v). Circles represents storage modulus data points. Light blue background corresponds to range of PNCM storage modulus (Fig S1).

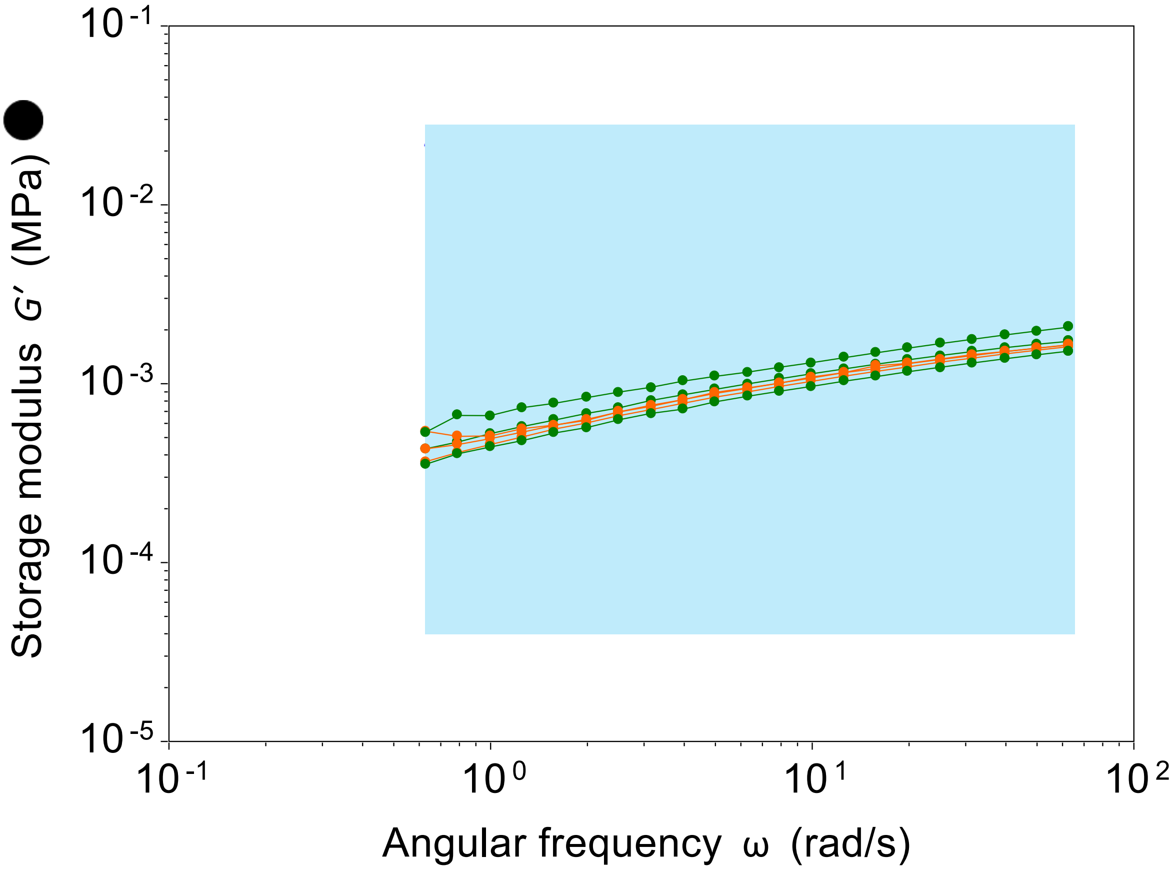

Figure S3. Storage modulus of PACM HEC-with two buffer systems. Green: PACM HEC BES pH 7.3; Orange: PACM HEC BES pH 6.3. Circles represents storage modulus data points. Measurements were performed in triplicates; each line represents single replicate. Choice of buffer system and pH did not affect the storage modulus.

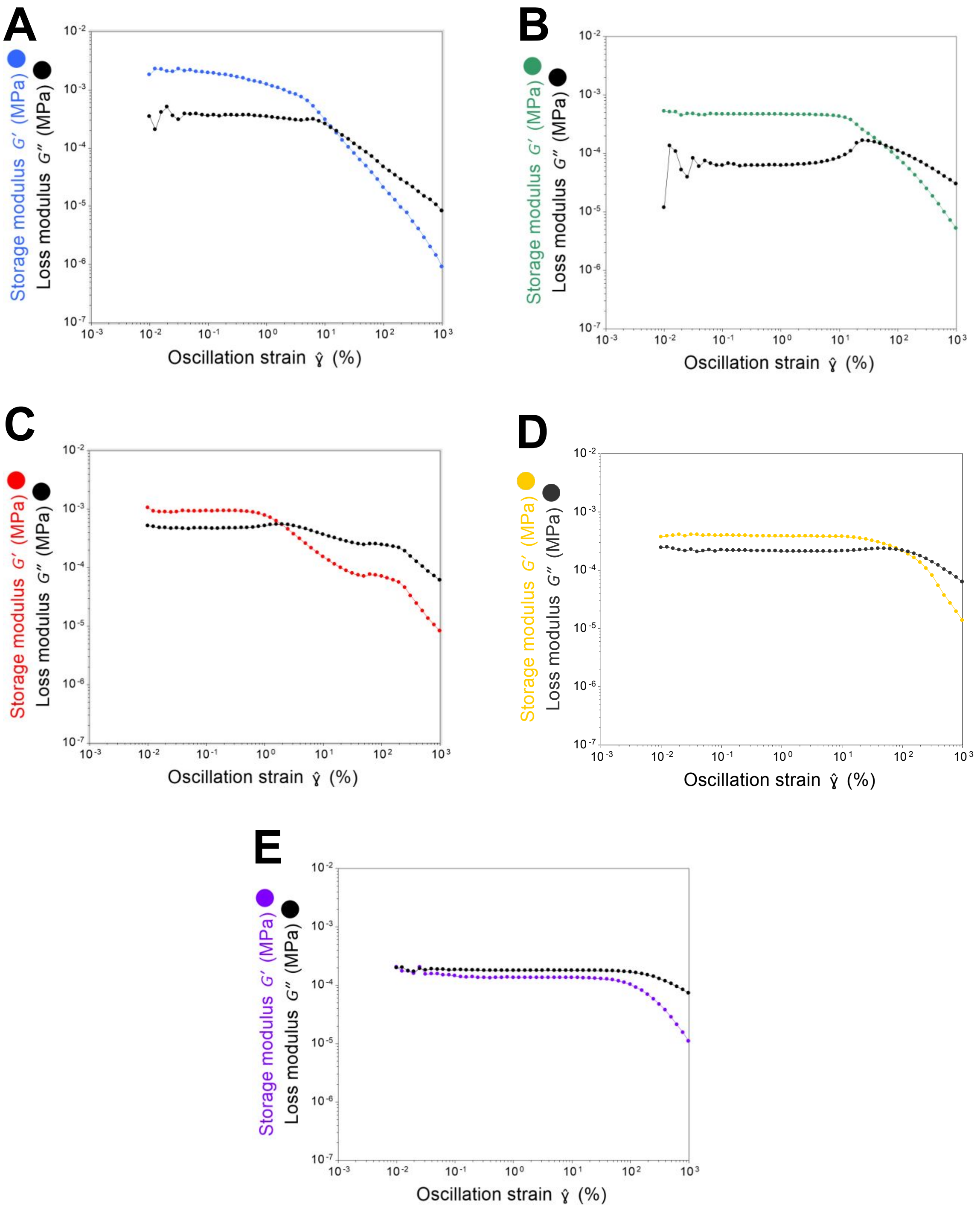

Figure S4. Amplitude sweep measurements showing viscoelastic behavior and the linear viscoelastic region. Black circles indicate the loss modulus; colored circles, the storage modulus. (A) native mucus; blue; (B) PACM PAA, green; (C) PACM HEC, red; (D) PACM SH, yellow; and (E) PACM SA, purple.

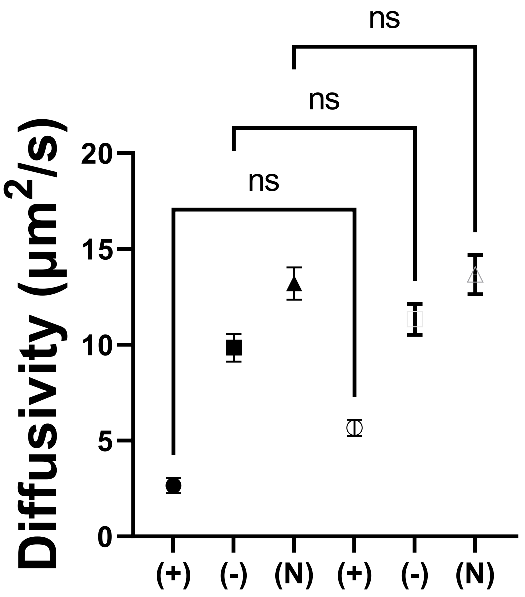

Figure S5. Diffusion of fluorescently labelled dextrans in artificial mucus (PACM). The FRAP diffusion measurements were performed with 4K cationic (+) in circle; anionic (-) in square; and neutral (N) in triangle; fluorescently-labeled dextrans in porcine artificial colonic mucus with polyacrylic acid (PACM PAA) presented by filled symbols and porcine artificial colonic mucus with hydroxyethyl cellulose (PACM HEC) presented by empty symbols. Measurements were performed in triplicates.

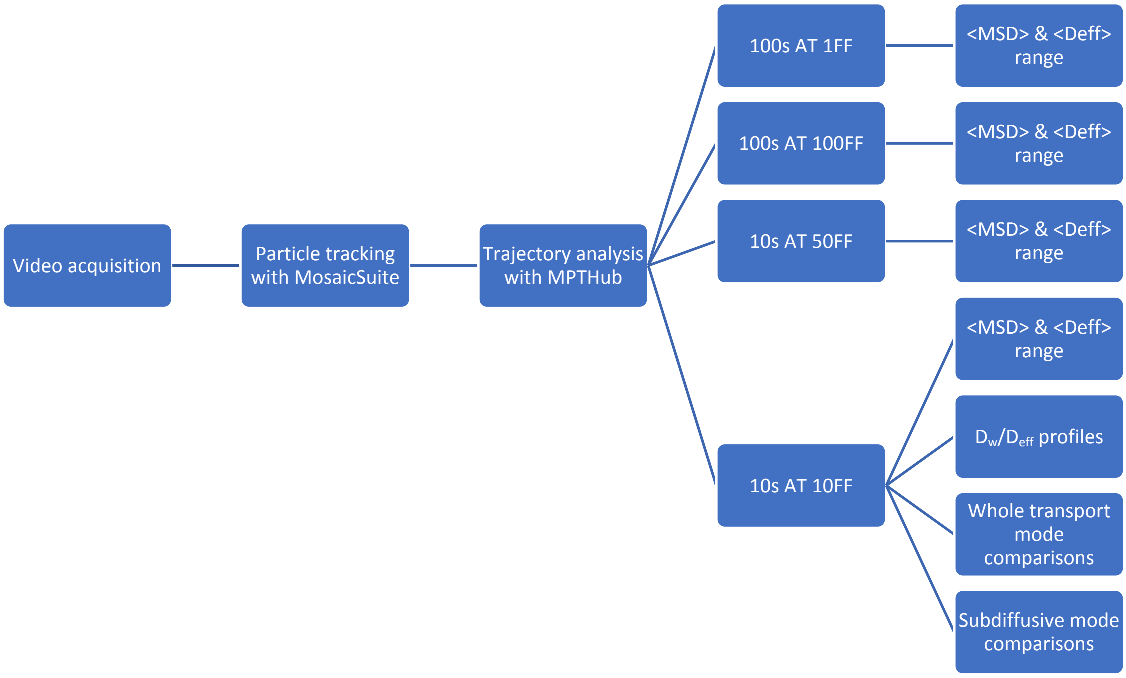

Figure S6. Workflow of video and trajectory data analyses to obtain accurate diffusion data. We explored transport mode of particles, mean squared displacement (MSD) range, effective diffusivity range, and hindrance of particles movement (D_w/ D_{eff}).

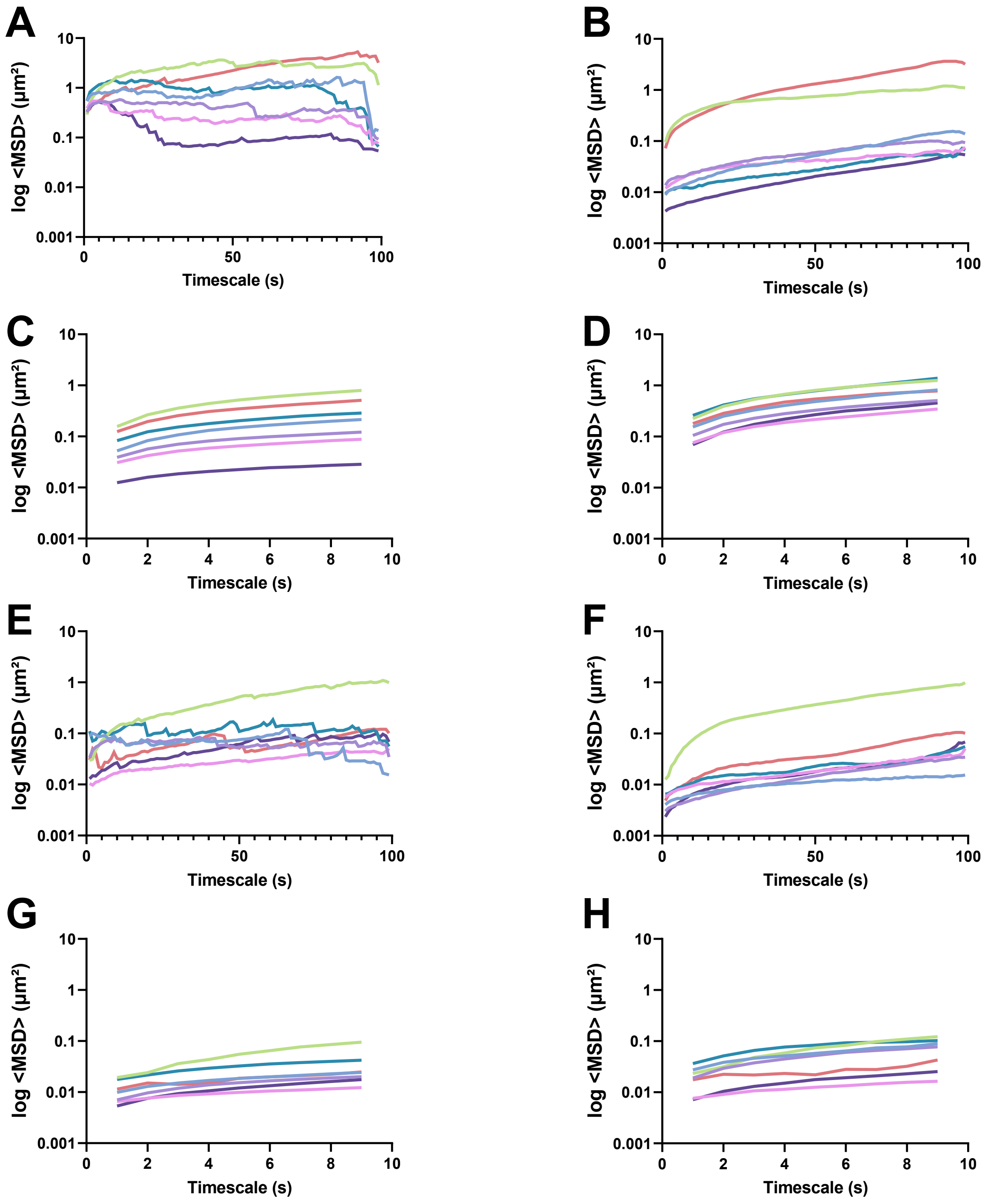
A
B
C
D
E
F
G
H
log <MSD> (µm²)
Timescale (s)
10
1
0.1
0.01
0.001
0
50
100
2
4
6
8

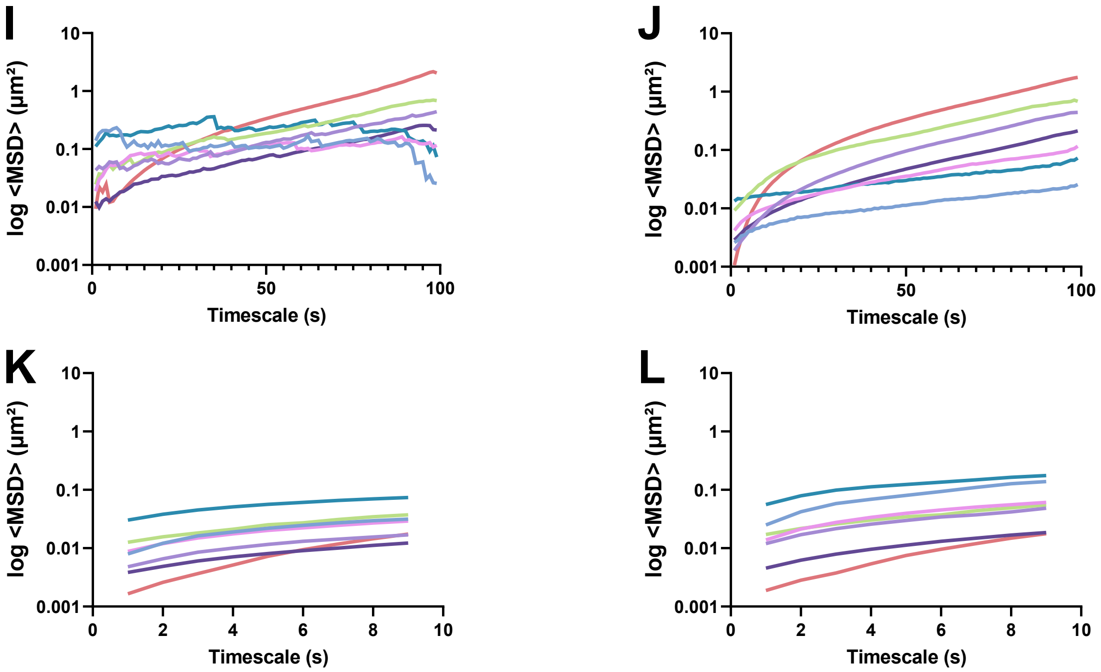

Figure S7. Average mean square displacement (MSD) ranges from different analysis times and frame filters for the three type of mucus (PNCM, PACM PAA, and PACM HEC). <D_{eff}> range from <MSD> between analysis time (AT) of 100s and 10s with frame filters (FF) of 1, 10, 50, and 100. A-D) PNCM; E-H) PACM PAA; I-L) PACM HEC. Light blue: 0.1 µm (-); Light purple: 0.2 µm (-); Pink: 0.5 µm (-); Green: 1 µm (-); Teal blue: 0.1 µm (+); Dark blue: 0.2 µm (+); Red: 1 µm (+). A, E, and I 100s AT with 1FF. B, F, and J 100s AT with 100FF. C, G, and K 10s AT with 50FF. D, H, and L 10s AT with 10FF. At least 100 particles were analyzed for each line.

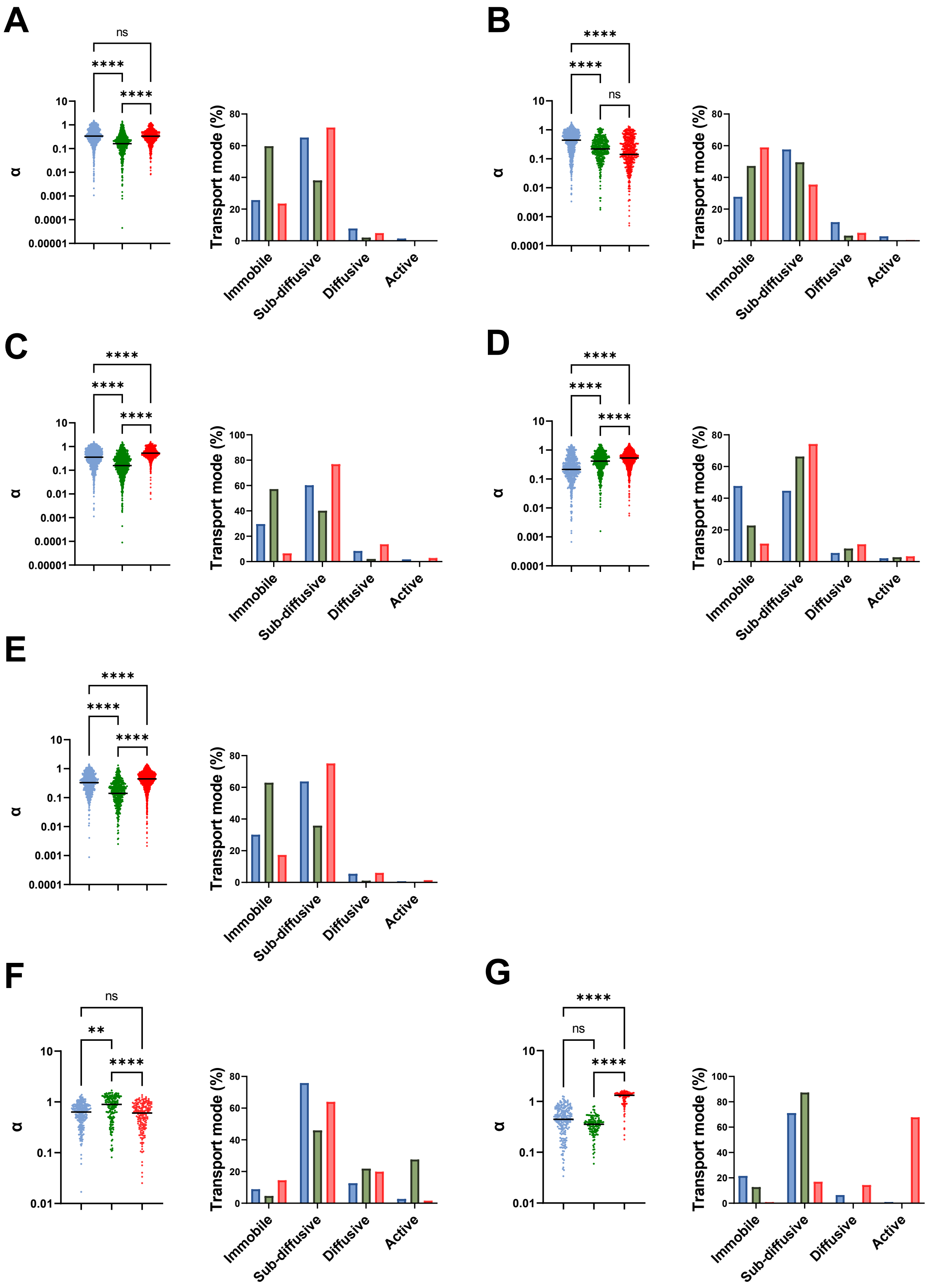

Figure S8. Anomalous coefficient distribution corresponding to the classification of particle movement (immobile, subdiffusive, diffusive, and active) in the three mucus types (PNCM, PACM PAA; PACM HEC). (A, C, E, F) Anionic polystyrene nanoparticles. (B, D, G) Cationic polystyrene nanoparticles. A) 0.1 μm (-); C) 0.2 μm (-); E) 0.5 μm (-); F) 1 μm (-); B) 0.1 μm (+); D) 0.2 μm (+); G) 1 μm (+). Blue: PNCM. Green: PACM PAA. Red: PACM HEC.

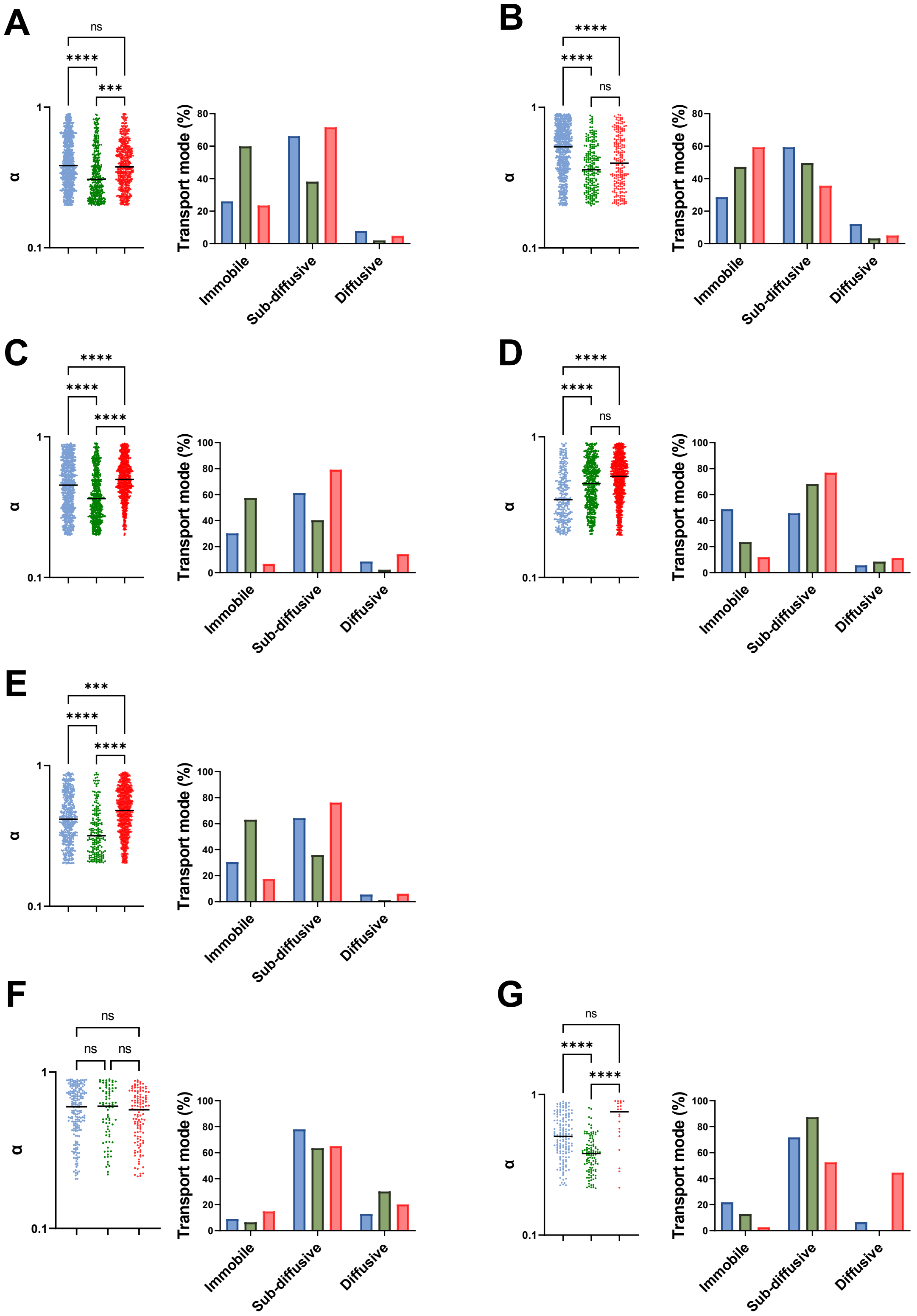

Figure S9. Anomalous coefficient distribution (only subdiffusive) which corresponds to classification of particles movement in the mucus (immobile, subdiffusive, and diffusive). Trajectories which classified as active diffusion was excluded from the analysis. (A, C, E, F) Anionic polystyrene nanoparticles. (B, D, G) Cationic polystyrene nanoparticles. A) 0.1 µm (-); C) 0.2 µm (-); E) 0.5 µm (-); F) 1 µm (-); B) 0.1 µm (+); D) 0.2 µm (+); G) 1 µm (+). Blue: PNCM. Green: PACM PAA. Red: PACM HEC.

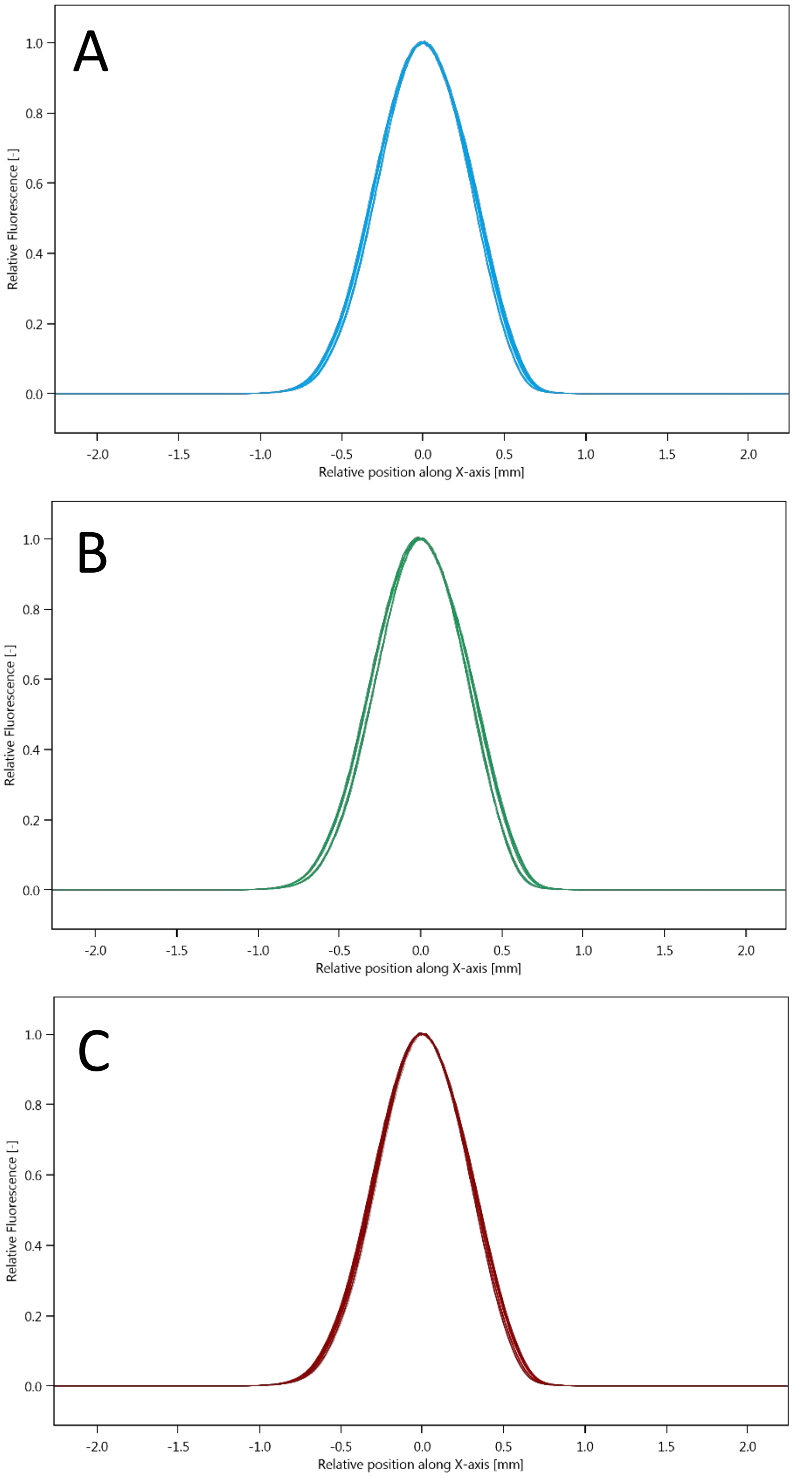

Figure S10. Capillary profiles of PACM and FITC-dextran interaction. The mean relative fluorescence is plotted vs. relative position along the x-axis. Plots show cationic 4K FITC-dextrans with diluted: A) PNCM (blue), B) PACM PAA (green), and C) PACM HEC (red). The shape of the curves is highly symmetrical, indicating minimal aggregation, which is ideal for MST measurement. Each experiment was performed in triplicates. Each curve represent one concentration of a serial dilution.

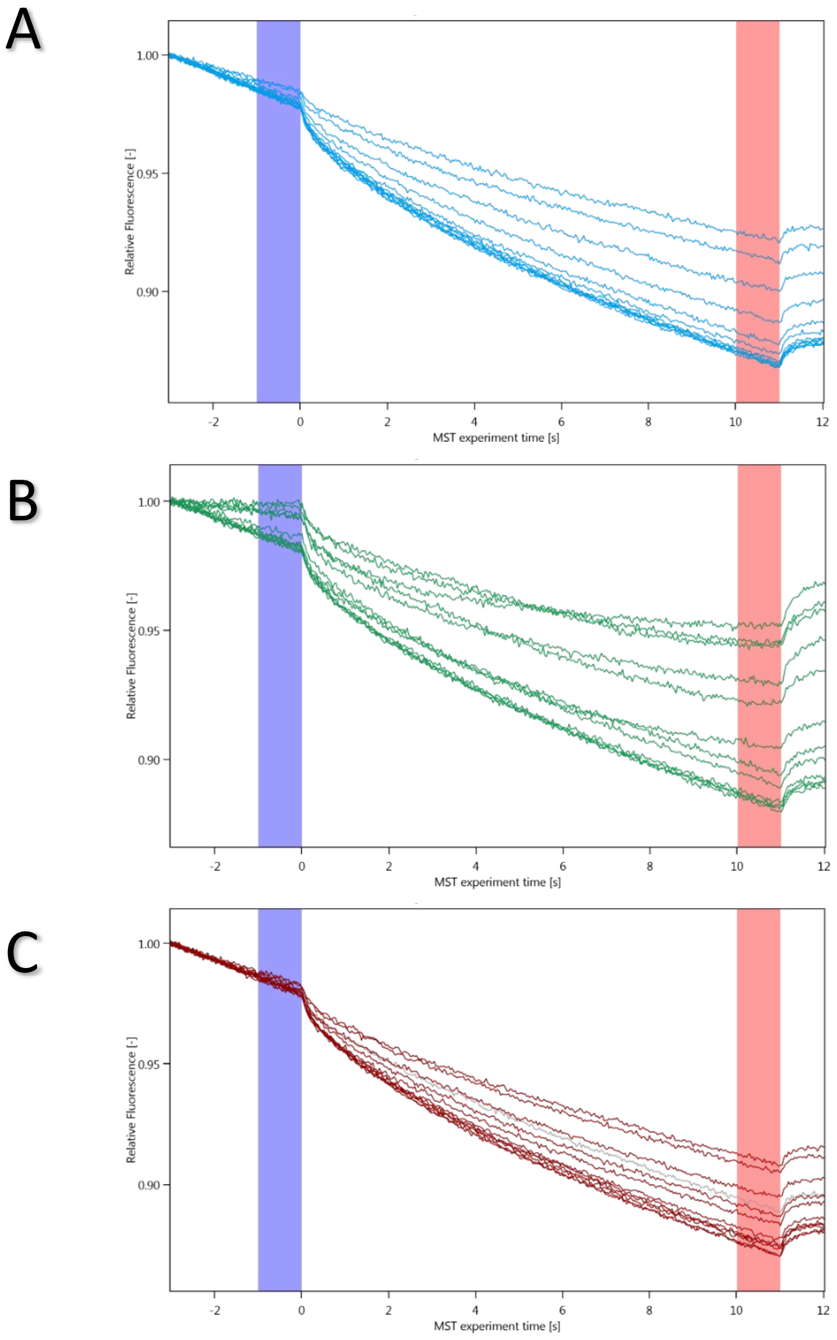

Figure S11. Microscale thermophoresis of 4K cationic FITC-dextran. FITC-dextran with serial dilutions of dispersed mucus in MES buffer as the target: A) PNCM, B) PACM PAA, C) PACM HEC. Only one of the three replicates is shown for clarity. Blue highlights the beginning before MST, while red highlights the endpoint.

Table S1. PACM core formulation preparation protocol

**Components**	**Quantity**	**Function**
Polymer	Optimal amount aiming to mimics PNCM 2-5% (w/v) of mucins	Gelling agent
Non-isotonic MES buffer	9 mL	Buffer
Porcine mucin type II	90.7 mg	Protein component
Bovine serum albumin	702 mg	Protein component
Lipid mixture	1 mL in total of the following mix	
- Isotonic MES buffer	- 1 mL	Buffer
- Cholesterol	- 23.09 mg	Lipid component
- Phosphatidylcholine	- 12.94 mg	Lipid component
- Tween 80	- 13 µL	Lipid solubilizer
Total amount	Approximate 10 mL	

Table S2. Hydrodynamic sizes and zeta potential of polystyrene nanoparticles in the particle tracking experiments. Measurements were performed in triplicates.

Type and particle size (nm)	Zeta-potential (mV)	SD (±)	Hydrodynamic sizes (nm)	SD (±)
Amine				
100	58.8	1.8	95.9	3.8
200	22.3	2.2	283.9	19.2
1000	-3.6	0.4	1315.9	467.7
Carboxylate				
100	-43.7	5.2	111.8	2.6
200	-51.8	2.5	237.5	13.5
500	-36.3	1.3	476.2	19.5
1000	-37.7	1.9	2440.8	1818.5

Table S3. Summary of differences between the two datasets of whole transport mode and sub-diffusive. Significances level were compiled from Fig. S8 and Fig. S9.

PNCM	PACM PAA		PACM HEC	
	whole transport mode	sub-diffusive	whole transport mode	sub-diffusive
Anionic				
0.1	****	****	NS	NS
0.2	****	****	****	****
0.5	****	****	****	***
1	**	NS	NS	NS
Cationic				
0.1	****	****	****	****
0.2	****	****	****	****
1	NS	****	****	NS

Table S4. Types of mucus systems with nanoparticle size and charge, average α (anomalous coefficient distribution), and diffusion in water (D_0) compared to diffusion in mucus (Dw/Deff) excluding active transport.

Mucus type	Particle types and sizes (μm)	α (±SD)	D_0 (μm^2-s^{-1})	D_W / D_{eff}
PNCM	Anionic			
	0.1	0.38 (0.32)	0.02274	289.0
	0.2	0.38 (0.34)	0.01409	233.2
	0.5	0.37 (0.28)	0.00961	136.7
	1.0	0.60 (0.29)	0.03493	18.8
	Cationic			
	0.1	0.44 (0.37)	0.03858	170.3
	0.2	0.26 (0.33)	0.01266	259.5
	1.0	0.46 (0.30)	0.02161	30.4
PACM PAA	Anionic			
	0.1	0.18 (0.27)	0.00249	2636.9
	0.2	0.20 (0.30)	0.00214	1533.3
	0.5	0.17 (0.26)	0.00046	2882.7
	1.0	0.70 (0.31)	0.00340	192.9
	Cationic			
	0.1	0.25 (0.31)	0.00285	2306.4
	0.2	0.42 (0.36)	0.00071	4643.0
	1.0	0.34 (0.18)	0.00119	553.7
PACM HEC	Anionic			
	0.1	0.36 (0.26)	0.00384	1710.4
	0.2	0.56 (0.28)	0.00135	2438.7
	0.5	0.45 (0.28)	0.00170	774.4
	1.0	0.55 (0.40)	0.00153	430.2
	Cationic			
	0.1	0.21 (0.36)	0.00490	1341.3
	0.2	0.53 (0.29)	0.00052	6357.0
	1.0	0.83 (0.28)	0.00049	1332.8
